# DNAJB12/14 redox switching directs chaperone- and Bax/Bak-dependent ER protein reflux

**DOI:** 10.1016/j.redox.2026.104324

**Published:** 2026-07-25

**Authors:** Laila Abu Madegam, Noa Gavriel, Raifu Tolulope Adebisi, Aeid Igbaria

**Affiliations:** The Department of Life Sciences, Ben-Gurion University, Beersheba, 841050, Israel

## Abstract

Maintenance of endoplasmic reticulum (ER) proteostasis is essential for cellular homeostasis and survival during stress. Beyond canonical quality control pathways, ER-to-cytosol signaling (ERCYS) enables the reflux of ER-resident proteins into the cytosol, where they can acquire noncanonical functions that promote cell survival. However, the mechanisms governing ERCYS and its relationship to ER stress remain poorly understood. Here, we show that ER protein reflux is restricted to a defined stress window and is governed by the ER redox environment. Mild ER stress maximizes protein reflux, whereas severe or reductive stress markedly suppresses this process. Mechanistically, we identify the ER-resident cochaperones DNAJB12 and DNAJB14 as redox-sensitive regulators of ERCYS. Under mild stress, intramolecular disulfide bonds stabilize DNAJB12 and DNAJB14, thereby supporting efficient protein reflux. In contrast, severe or reductive stress increases intracellular glutathione, reducing these disulfide bonds and promoting degradation of DNAJB12 and DNAJB14, resulting in the loss of chaperone-mediated reflux. We further show that protein reflux requires cysteine-dependent interactions between refluxed substrates and the cytosolic cochaperone SGTA, revealing a previously unrecognized redox-sensitive step in the ERCYS pathway. When ERCYS is impaired during severe ER stress, cells instead engage an alternative apoptosis-associated pathway mediated by BAX/BAK-dependent ER membrane permeabilization. This transition is driven by enhanced recruitment of BAX and BAK to the ER by the BH3-only protein BIK, amplifying apoptotic signaling. Together, these findings establish redox regulation as a molecular switch that determines whether cells mount an adaptive ER protein reflux response or commit to BAX/BAK-dependent ER membrane permeabilization and apoptosis.

## 1 Introduction

The endoplasmic reticulum (ER) is the primary site for the folding and maturation of secretory and membrane proteins. Within the ER, oxidative protein folding is essential for their structural maturation and functional integrity, as it catalyzes the formation of disulfide bonds in nascent polypeptides [1]. These covalent linkages stabilize higher-order protein structures, facilitate correct protein folding, and enhance protein stability [[2], [3], [4]]. Because nearly one-third of the cellular proteome transits through the secretory pathway, the fidelity of oxidative protein folding is critical for maintaining proteome integrity and cellular homeostasis [[5], [6], [7]].

To sustain its high biosynthetic flux, the ER relies on tightly regulated quality control systems. Central to this is the unfolded protein response (UPR), which enhances ER folding capacity while simultaneously reducing protein load under conditions of proteotoxic stress [[8], [9], [10], [11], [12]]. The UPR promotes selective degradation of mRNAs encoding ER-targeted proteins, attenuates global protein translation, and coordinates downstream clearance pathways [[13], [14], [15]]. Misfolded or unassembled proteins are eliminated via the ER-associated degradation (ERAD), which mediates retrotranslocation to the cytosol and subsequent proteasomal degradation [16,17]. Additional adaptive mechanisms include autophagic removal of damaged ER subdomains (ER-phagy), activation of a pre-emptive quality-control pathway that redirects newly synthesized ER-targeted proteins to the cytosol for degradation during stress, and reflux of ER-resident proteins into the cytosol [[16], [17], [18], [19], [20], [21], [22], [23], [24]]. These pathways operate in a coordinated, highly interconnected manner, with extensive crosstalk among them, collectively preserving ER proteostasis and preventing the accumulation of cytotoxic protein species that threaten cellular viability [17].

In addition to canonical stress pathways, ER protein load can be alleviated through ER-to-Cytosol Signaling (ERCYS) [17,19,20]. This stress-induced, chaperone-mediated mechanism redistributes ER-resident proteins to the cytosol. ERCYS is constitutively active in cancer cells and promotes the cytosolic enrichment of ER proteins in cultured tumor cells, murine brain tumor models, and patient samples. Once relocalized, these proteins acquire noncanonical, pro-survival functions that enhance tumor cell fitness, including inhibition of wild-type p53 and caspase-3 activity. ERCYS is regulated by ER and cytosolic chaperones, including DNAJB12, DNAJB14, and the HSC70 cofactor SGTA, whose depletion abolishes ER protein reflux [12,[25], [26], [27]].

Stress-induced spatial redistribution is not confined to the ER but extends to multiple organelles, including mitochondria, peroxisomes, lysosomes, and the nucleus, resulting in global subcellular proteome remodeling that is not captured by changes in protein abundance. However, the signaling pathways governing this process remain poorly defined [[28], [29], [30], [31]].

Recently, we identified a previously unrecognized role for the UPR in orchestrating ERCYS, with central contributions from ATF6 and IRE1 signaling. IRE1 exerts dual control by promoting ER protein reflux while limiting BAX/BAK-mediated membrane permeabilization. We further demonstrate that BiP functions as a bidirectional regulator of ER protein trafficking by assembling into a stress-inducible complex with membrane-anchored and cytosolic cochaperones [12]. These findings redefine BiP beyond its canonical role in protein import and establish UPR-driven spatial proteome remodeling as a critical mechanism of stress adaptation.

ER protein redistribution is believed to be redox-driven; however, the underlying molecular mechanisms remain poorly defined [23]. Regulation of DNAJB12 and DNAJB14 involves thiol-based redox processes that may influence their stability and physiological function. Here, we demonstrate that increasing ER stress negatively correlates with protein reflux in a dose-dependent manner. We further show that transient disulfide bridges form between the refluxed PDI proteins and cytosolic HSC70-cochaperone SGTA near the ER membrane, supporting a redox-sensitive trafficking mechanism. Elevated ER stress or DTT treatment, increase cellular glutathione pool, break the intradisulfide bridge in DNAJB12/14 and accelerates degradation of DNAJB12 and DNAJB14, thereby suppressing ERCYS. This redox-dependent decrease in DNAJB12/14 levels shifts the cellular response toward ERCYS-independent ER protein permeabilization mediated by the proapoptotic BCL-2 homology (BH3)-only ER-resident protein BCL-2 interacting killer (BIK) that recruits BAX/BAK to the ER membrane to reflux proteins to the cytosol, a process associated with apoptotic signaling. Together, these findings suggest that redox regulation functions as a molecular switch between adaptive protein reflux and pro-apoptotic membrane permeabilization.

## 2 Results

### 2.1 I. ER to cytosol reflux is attenuated during severe stress

To determine the stress threshold at which ERCYS is initiated and to define its maximal extent, we exposed cells to increasing concentrations of mechanistically distinct ER stressors. These included tunicamycin (Tm), which inhibits N-linked glycosylation; thapsigargin (Tg), a potent inhibitor of the SERCA Ca^2+^ pump; and dithiothreitol (DTT), a strong reducing agent that disrupts disulfide bond formation.

**Fig. 1:**
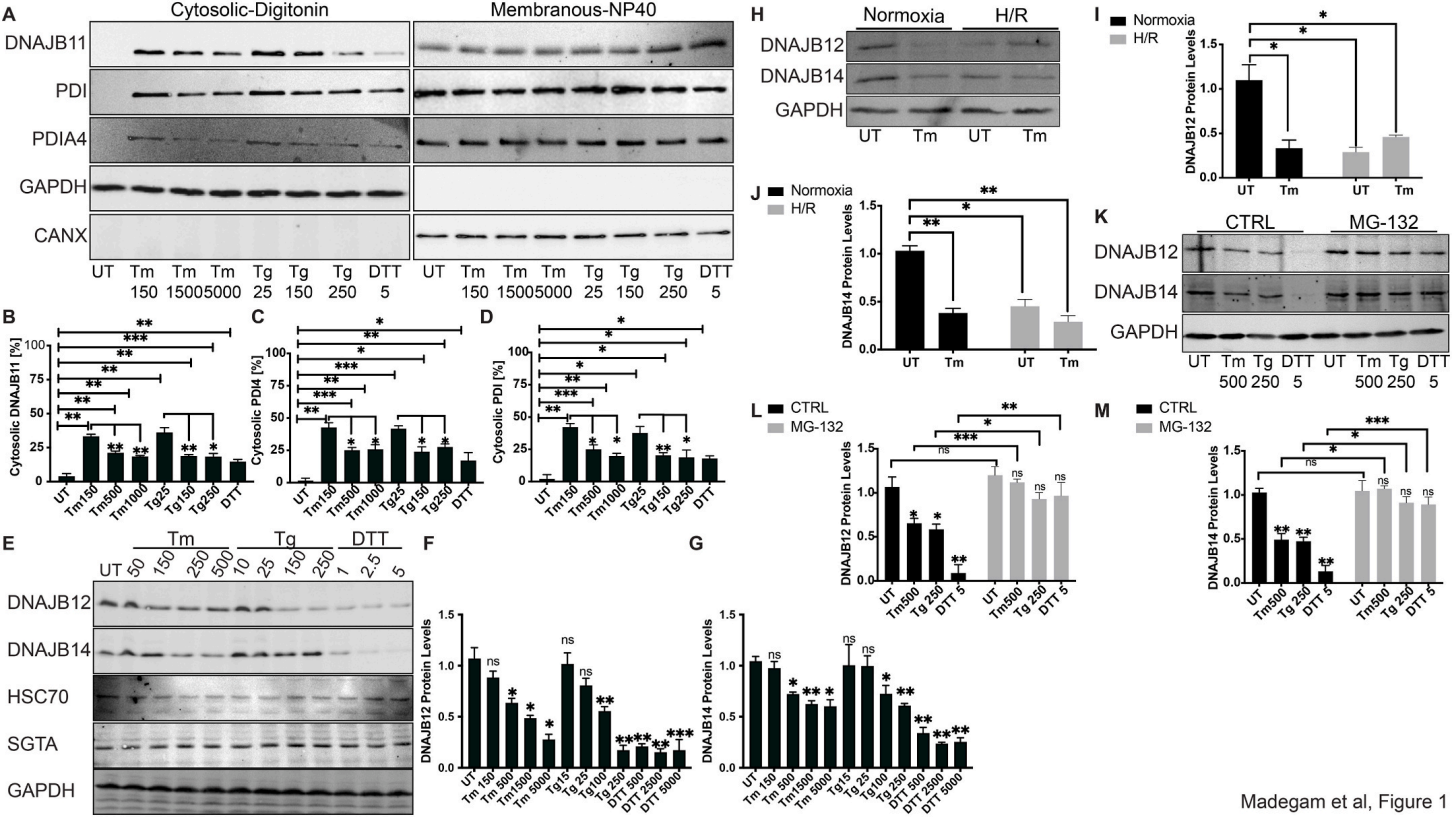
Stress-induced degradation of DNAJB12/14 limits ER protein reflux. (A) Subcellular protein fractionation (Cytosolic-Digitonin fraction) of DNAJB11, PDIA1 (PDI), and PDIA4 in A549 cells treated with Tm (ng/mL), Tg (nM), and DTT (mM). N = 3. **(B**–**D)** Quantification of the refluxed ER proteins DNAJB11, PDIA1 (PDI), and PDIA4 as in A, respectively. **(E)** Representative immunoblot of DNAJB12, DNAJB14, HSC70, SGTA, and GAPDH in A549 cells treated with Tm (ng/mL), Tg (nM), and DTT (mM) as indicated. N = 3. **(F**–**G)** Quantification of DNAJB12(F) and DNAJB14(G) as shown in E and normalized to GAPDH. **(H)** Representative immunoblot of DNAJB12, DNAJB14, and GAPDH in H9C2 cells treated with Tm (ng/mL), after hypoxia/reoxygenation (H/R) as indicated. **(I**–**J)** Quantification of DNAJB12(I) and DNAJB14(J) as shown in H and normalized to GAPDH N = 3. **(K)** Representative immunoblot of DNAJB12, DNAJB14, and GAPDH in A549 cells treated with Tm (ng/mL), Tg (nM), and DTT (mM) in the presence of the proteasome inhibitor MG-132. **(L**–**M)** Quantification of DNAJB12(L) and DNAJB14(M) as shown in K and normalized to GAPDH N = 3. All experiments were done in biological triplicates. (***p < 0,001, **p < 0,01, *<0.05).

We monitored the reflux using several ER-resident proteins, including protein disulfide isomerase A1 and A4 (PDIA1 and PDIA4) and the DnaJ homolog subfamily B member 11 (DNAJB11), as representative reporters of the process. We treated A549, HEK293T and MDA-MB-231 cells with increasing concentrations of Tm, Tg, or DTT, followed by cell harvesting and subcellular fractionation to assess protein redistribution between the ER and the cytosol. Contrary to our expectation that increasing ER stress would proportionally enhance reflux, we observed that low concentrations of Tm and Tg induced maximal reflux, reaching approximately ∼42% cytosolic redistribution of the monitored ER proteins (Fig. 1A–D and Fig. S1A–D). At higher concentrations, reflux persisted but was significantly attenuated, with cytosolic levels reduced to approximately 15-25%. Thus, reflux efficiency did not scale linearly with stress intensity (Fig. 1A–D and Fig. S1A–D). Moreover, DTT at all concentrations barely induced the reflux of proteins to the cytosol despite a potent UPR activation (Fig. 1A–D and Fig. S1A–J). We validated those findings in HEK293T cells treated with different concentrations of Tm, Tg and DTT. At low concentrations, HEK293T cells displayed robust ER protein reflux that was attenuated under higher levels of ER stress or DTT treatemnet (Fig. S1K–N).

To investigate whether ERCYS occurs under physiologically relevant conditions, we utilized a hypoxia-reoxygenation (H/R) model in H9C2 cells, known to induce simultaneous ER stress. Upon reoxygenation, we observed that only a small fraction of DNAJB11 relocalized to the cytosol, whereas PDI and PDIA4 remained sequestered within the ER. In contrast, treatment with low-dose Tm under normoxic conditions triggered the robust relocalization of all tested proteins. Notably, this reflux was significantly attenuated or inhibited when Tm treatment was combined with H/R conditions, despite the induction of the UPR at levels comparable to those observed under normoxia (Fig. S1O–S). These findings indicate that ERCYS is optimally activated within a specific stress window and is suppressed under conditions of excessive or compounding stress.

We next examined whether the attenuation of reflux observed at higher stressor concentrations could be attributed to reduced cell viability following short-term treatment. To address this possibility, we quantified cell survival and apoptotic activation under the same experimental conditions. Although elevated concentrations of tunicamycin and thapsigargin led to slight caspase-3 activation, the overall number of viable cells remained largely unchanged during the 8-h exposure period (Fig. S2A–B). These findings indicate that some cells were in the early stages of apoptosis and had not yet undergone substantial cell loss. Therefore, the reduced reflux observed at higher stress levels cannot be explained by decreased cell viability.

Collectively, these results support a model in which ER protein reflux represents an adaptive mechanism that operates optimally within a defined, intermediate stress window. When stress exceeds this threshold and apoptotic signaling begins to dominate, ERCYS is suppressed, and alternative ERCYS-independent pathways, may predominate, as exemplified by the response to DTT.

### 2.2 Reductive stress attenuates ER protein reflux by destabilizing DNAJB12/14

DTT is a well-established inducer of ER stress by reducing disulfide bonds in the ER lumen (Fig. S1E–J). Beyond its effects on protein folding, DTT has been shown to selectively destabilize DNAJB12 by promoting its proteasomal degradation [23,32]. We therefore propose that the attenuation of protein reflux observed under severe ER stress reflects an active, stress-dependent limitation of the ERCYS machinery rather than a passive consequence of overwhelming misfolded protein load. Consistent with this model, we examined the abundance of ERCYS components following exposure to increasing concentrations of tunicamycin, thapsigargin, or DTT, enabling us to assess whether stress severity differentially impacts the stability of DNAJB12 and DNAJB14 relative to other chaperone partners. DNAJB12 and DNAJB14 protein levels remained stable at low concentrations of Tm and Tg, coinciding with maximal reflux activity. In contrast, treatment with DTT resulted in a pronounced reduction of DNAJB12 and DNAJB14 protein levels at all tested concentrations (Fig. 1E–G). At high concentrations of Tm and Tg, DNAJB12 and DNAJB14 were nearly undetectable, indicating that severe ER stress or reductive stress leads to loss of the DNAJB12 and DNAJB14 cochaperones. In contrast, levels of SGTA and HSC70 remained unchanged across all conditions tested (Fig. 1E–G, and Fig. S2C–D), suggesting selective sensitivity of DNAJB proteins to stress intensity and redox state.

**Fig. 2:**
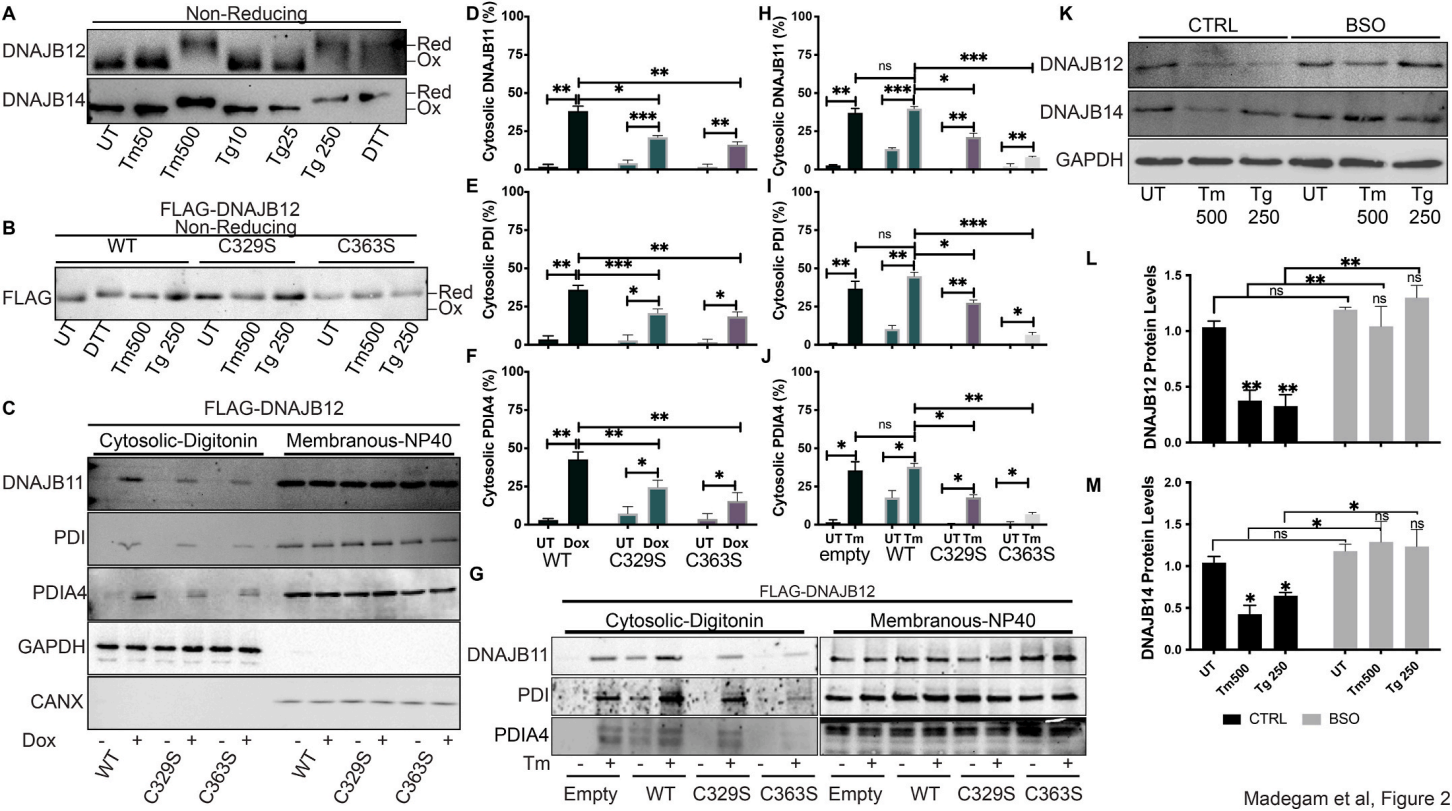
Reduction of the intramolecular disulfide bonds in DNAJB12 and DNAJB14 impairs ERCYS. (A) Representative non-reducing immunoblot showing NEM-labeled DNAJB12 and DNAJB14 in A549 cells treated with Tm (ng/mL), Tg (nM), and DTT (mM). Reduced and oxidized protein species are indicated. N = 3. **(B)** Representative non-reducing immunoblot of NEM-labeled FLAG-tagged DNAJB12 WT and cysteine mutants (C329S and C363S) in A549 cells treated with Tm (ng/mL) and Tg (nM). Both reduced and oxidized protein forms are shown. N = 3. **(C)** Subcellular protein fractionation showing DNAJB11, PDIA1 (PDI), and PDIA4 in A549 cells expressing FLAG-tagged DNAJB12 WT or cysteine mutants (C329S and C363S) under a doxycycline-inducible promoter. N = 3. **(D**–**F)** Quantification of the refluxed ER proteins DNAJB11, PDIA1 (PDI), and PDIA4 as in C, respectively. **(G)** Subcellular protein fractionation showing DNAJB11, PDIA1 (PDI), and PDIA4 in A549 cells expressing FLAG-tagged DNAJB12 WT or cysteine mutants (C329S and C363S) in the presence of Tm. N = 3. **(H**–**J)** Quantification of the refluxed ER proteins DNAJB11, PDIA1 (PDI), and PDIA4 as in G, respectively. All experiments were done in biological triplicates. **(K)** Representative immunoblot showing DNAJB12, DNAJB14, and GAPDH in A549 cells treated with Tm (ng/mL) and Tg (nM) in the presence of l-Buthionine-sulfoximine (BSO). **(L**–**M)** Quantification of DNAJB12(L) and DNAJB14(M) as shown in K and normalized to GAPDH N = 3. All experiments were done in biological triplicates. (***p < 0,001, **p < 0,01, *<0.05).

In the hypoxia-reoxygenation model, both DNAJB12 and DNAJB14 underwent substantial degradation (Fig. 2H–J). Our results demonstrate that H/R induces marked degradation of DNAJB12, which is associated with inhibition of ER protein reflux (Fig. S1O–S).

To investigate the mechanism underlying DNAJB12 and DNAJB14 degradation and determine whether this process is proteasome-dependent, cells were treated with the proteasome inhibitor MG-132. Under these conditions, both DNAJB12 and DNAJB14 were stabilized even under high stress conditions, indicating that their degradation is mediated by the proteasome (Fig. 1K–M and Fig. S2E–G). Collectively, these findings demonstrate that DNAJB12 and DNAJB14 undergo proteasome-dependent degradation in response to severe ER stress, reductive stress, and hypoxia-reoxygenation, supporting a potential role for the loss of these co-chaperones in the impairment of ER protein reflux.

Although DNAJB12 was degraded under DTT-induced reductive stress, approximately 25% of ER proteins still escaped the ER (Fig. 1A–J). We therefore asked whether ER stress induced by high levels of Tm and Tg similarly alters the ER redox state, as observed with DTT, and consequently inhibits protein reflux through a comparable mechanism. For this, we pretreated cells with DTT prior to low levels of Tm and Tg treatment. In those conditions, DTT preatement decreased the reflux of proteins from the ER to the cytosol (Fig. S3A–H). Interestingly, despite this, still ER protein managed to escape the ER to the cytosol suggesting an alternative route to be activated under reductive stress and is activated independently of DNAJB12. This selective vulnerability of DNAJB12 suggests that it may function as a regulatory checkpoint within the ER-to-cytosol reflux pathway. Because DNAJB12 is a core component of the ERCYS-associated chaperone complex, together with DNAJB14, HSC70, and SGTA, its preferential loss under high-stress conditions may selectively turn off reflux. Such a mechanism would allow cells to engage reflux transiently under moderate stress, but suppress it when ER dysfunction becomes severe.

To determine whether the degradation of DNAJB12 and DNAJB14 is driven by reductive stress and concomitant changes in the redox state of their ER-luminal cysteines [23], we monitored the redox status of both proteins. Under basal and mild stress conditions, alkylation with NEM followed by non-reducing SDS-PAGE revealed that DNAJB12 and DNAJB14 migrated faster than their fully reduced counterparts, consistent with an oxidized conformation containing intramolecular disulfide bonds that promote a compact structure (Fig. 2A and Fig. S4A). Conversely, treatment with high concentrations of tunicamycin or thapsigargin induced a pronounced shift toward slower-migrating species. These shifted bands closely resembled the migration pattern observed under DTT treatment, indicating a transition to a reduced state (Fig. 2A and Fig. S4A). Notably, these reduced species exhibited a lower steady-state abundance, suggesting that the reduced conformation possesses inherently decreased stability compared to the oxidized form.

To identify the specific disulfide bonds involved in this redox transition, we generated serine substitution mutants (C329S and C363S) targeting the two cysteine residues predicted to form an intramolecular disulfide bond [23] (Fig. S4B–C). Under non-reducing conditions, both single-point mutants displayed gel migration patterns identical to DTT-treated wild-type samples, confirming a constitutively reduced state (Fig. 2B). Furthermore, these cysteine mutants exhibited reduced stability relative to wild-type DNAJB12 (Fig. S4B–C). Taken together, these data indicate that the integrity of these specific luminal cysteines and the formation of the intramolecular disulfide bond are critical structural requirements for maintaining DNAJB12 and DNAJB14 stability.

We previously showed that overexpression of wild-type DNAJB12 is sufficient to promote ER protein reflux independently of ER stress or UPR activation [26]. In agreement with this, overexpression of wild-type DNAJB12 robustly enhanced protein reflux to the cytosol. In contrast, both cysteine mutants failed to induce massive reflux compared to the WT, demonstrating that these residues are essential for DNAJB12 function (Fig. 2C–F). When tested under low ER stress conditions, wild-type DNAJB12 modestly enhanced reflux in response to low-dose tunicamycin, whereas the cysteine mutants, particularly C363, exerted a dominant-negative effect, suppressing reflux even in the presence of stress (Fig. 2G–J).

Together, these findings indicate that intramolecular disulfide bond formation is critical for DNAJB12 stability and activity. Under mild ER stress, the ER environment likely remains sufficiently oxidizing to preserve these disulfide bonds and sustain protein reflux. In contrast, under more severe or reductive conditions, disruption of these bonds leads to DNAJB12 destabilization and inhibition of reflux (Fig. 2A–J and Fig. S4A–C).

Consistent with previous findings that overexpression of DNAJB12 or its yeast orthologue HLJ-1 impair cell proliferation and promotes cell death in a J-domain-dependent manner [19,21,26], we examined the impact of cysteine residues on this phenotype. We observed that overexpression of DNAJB12 cysteine mutants resulted in significantly reduced cytotoxicity compared to the wild-type protein (Fig. S4D). These results suggest that the toxicity associated with DNAJB12 overexpression is inherently linked to its reflux activity. As excessive ER protein reflux appears to be deleterious, this process likely operates within a narrow adaptive window; thus, the reduction of DNAJB12 cysteines may serve as a regulatory mechanism to terminate reflux and limit cellular damage during periods of sustained stress.

To investigate the molecular switch governing the transition from ERCYS activation to its termination under severe ER stress, we examined factors that may change the ER redox environment. Because glutathione (GSH) serves as the major reducing equivalent in the ER by donating electrons during oxidative protein folding, we quantified total GSH levels and the GSH/GSSG ratio following ER stress induction. Exposure to high concentrations of Tm or Tg, conditions that terminate ERCYS, resulted in a significant increase in total GSH levels accompanied by an elevated GSH/GSSG ratio, indicating a shift toward a more reducing environment (Fig. S4E and F). To directly assess whether GSH contributes to the regulation of DNAJB12/14 stability, we depleted intracellular GSH by treating cells with BSO, an inhibitor of GSH biosynthesis. GSH depletion markedly stabilized both DNAJB12 and DNAJB14 under severe ER stress conditions, demonstrating that GSH promotes the degradation of these ER co-chaperones and implicating ER reducing power as a key regulator of the molecular switch that terminates ERCYS without affecting the severity of the stress (Fig. 2K–M, and Fig. S4G).

### 2.3 SGTA-mediated thiol-dependent substrate capture defines a cytosolic checkpoint for ERCYS

Beyond ER-resident factors, our data identify a critical cytosolic, redox-dependent checkpoint mediated by the co-chaperone SGTA. SGTA has been proposed to engage refluxed proteins through transient, cysteine-dependent disulfide bonds [26]. Given that DTT-induced reducing conditions inhibit ERCYS, we hypothesized that this inhibition stems from the disruption of essential cysteine-dependent interactions between SGTA and its substrates.

**Fig. 3:**
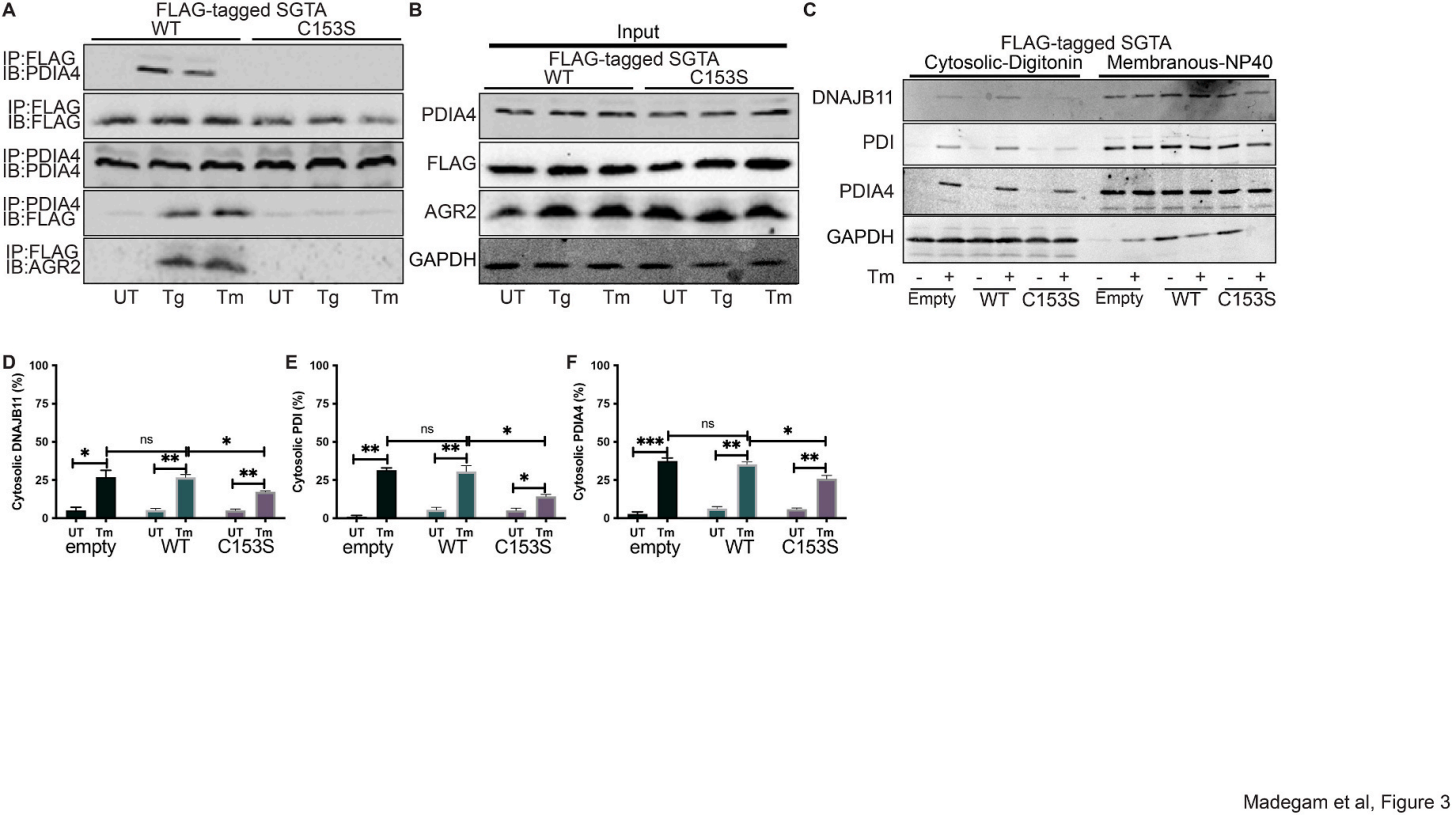
Redox-dependent signaling between cytosolic and ER proteins controls ER protein reflux. (A) Representative immunoblot demonstrating the interaction between FLAG-tagged SGTA WT and the C153S cysteine mutant with PDIA4 in A549 cells treated with tunicamycin or thapsigargin. N = 3. **(B)** Representative immunoblot of total protein lysates corresponding to the experiment shown in (A). **(C)** Subcellular fractionation analysis of DNAJB11, PDIA1 (PDI), and PDIA4 in A549 cells expressing FLAG-tagged SGTA WT or the C153S mutant under tunicamycin treatment. N = 3. **(D**–**F)** Quantitative analysis of refluxed ER proteins DNAJB11, PDIA1 (PDI), and PDIA4, respectively, as shown in (C). All experiments were performed in biological triplicates. Statistical significance is indicated as follows: *p < 0.05, **p < 0.01, ***p < 0.001.

To investigate the role of SGTA-mediated thiol interactions, we generated an SGTA C153S mutant targeting the conserved cysteine predicted to mediate disulfide bond formation with refluxed substrates such as AGR2. We then expressed FLAG-tagged WT-SGTA or the C153S variant and examined their ability to associate with refluxed proteins. Whereas WT SGTA efficiently interacted with the refluxed substrates PDIA4 and AGR2, the C153S mutant failed to bind either protein (Fig. 3A and B and Fig. S5A and B), demonstrating that cysteine 153 is essential for substrate association. Consistent with a functional requirement for this interaction during ERCYS, cytosolic accumulation of refluxed proteins was readily detected in control cells and in cells overexpressing WT SGTA but was markedly reduced in cells expressing the C153S mutant (Fig. 3C–F). These findings suggest that the C153S mutant acts in a dominant-negative manner by disrupting productive substrate capture following ER exit.

To further substantiate the requirement for thiol-dependent interactions, we generated an AGR2 C81S mutant, targeting the conserved cysteine predicted to facilitate cytosolic interaction with SGTA [26]. We found that the AGR2 C81S mutant failed to exit the ER under low-stress conditions that typically trigger ERCYS, unlike wild-type substrates such as PDIA4, which exited efficiently (Fig. S5C–E). This suggests that specific cysteine residues are required to license protein reflux, potentially serving as a selective checkpoint for ER exit. Notably, at high stress concentrations, AGR2 exited the ER regardless of this mutation, indicating that severe stress can trigger alternative, ERCYS-independent pathways for substrate export.

Collectively, these findings establish that ERCYS is governed by redox-regulated thiol chemistry at multiple stages: DNAJB12 orchestrates the initiation of reflux within the ER, whereas SGTA facilitates the capture of refluxed substrates in the cytosol via cysteine-dependent interactions. Together, these results define a coordinated, thiol-dependent mechanism as a fundamental regulatory axis for efficient ER protein reflux.

### 2.4 PDIA4 gain of function depends on the active cysteine in Capsae-3

Upon reflux to the cytosol, PDIA4 redistributes and engages in inhibitory interactions with caspase-3, as previously reported [27]. Caspase-3 is a cysteine-aspartic protease whose activity critically depends on cysteine 163 within its catalytic site, and its function can be suppressed by redox-based modifications such as S-nitrosylation [33,34].

**Fig. 4:**
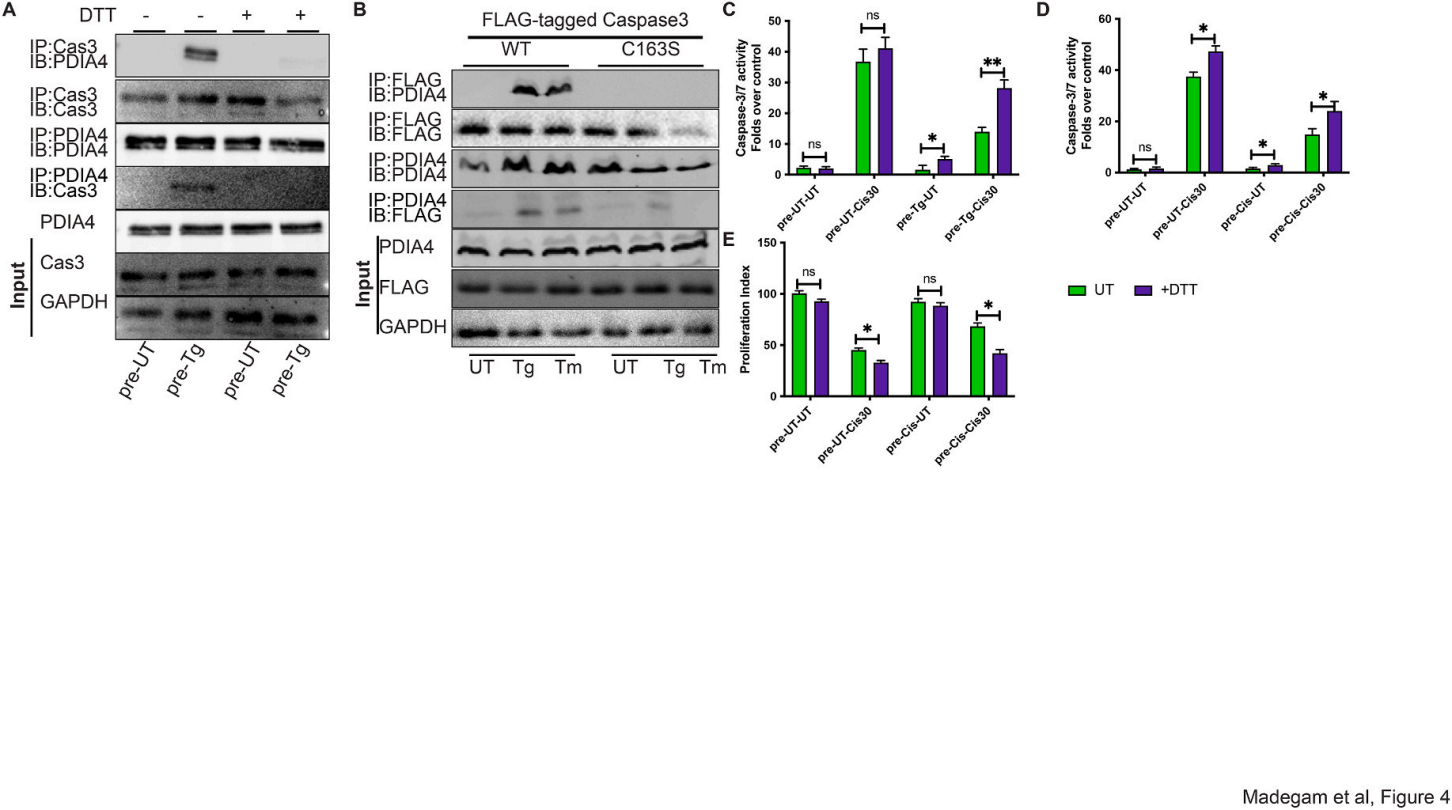
Active site cystene in caspase-3 is necessary for its activity and interaction with PDIA4. (A) Representative immunoblot showing the interaction between caspase-3 and PDIA4 in chemoresistant A549 cells pre-treated with thapsigargin (Tg) and DTT. N = 3. **(B)** Representative immunoblot demonstrating the association of FLAG-tagged caspase-3 WT or the C163S mutant with PDIA4 following treatment with tunicamycin (Tm) or thapsigargin (Tg). N = 3. **(C)** Fold change in caspase-3/7 activity in chemoresistant A549 cells pre-treated with thapsigargin and subsequently exposed to the indicated concentrations (μM) of cisplatin. N = 3. **(D)** Fold change in caspase-3/7 activity in chemoresistant A549 cells pre-conditioned with low-dose cisplatin and then treated with increasing concentrations (μM) of cisplatin. N = 3. **(E)** XTT viability assay in cisplatin-pretreated A549 cells challenged with higher concentrations (μM) of cisplatin, as indicated. N = 3. All experiments were conducted in biological triplicates. Statistical significance is denoted as follows: *p < 0.05, **p < 0.01, ***p < 0.001.

Chronic exposure to low-level Thapsigargin for three days promoted cancer cell resistance by facilitating the relocalization of PDIA4 to the cytosol, where it binds to and inhibits caspase-3 activity [27]. Consistent with this, we observed an enhanced interaction between PDIA4 and caspase-3 in these resistant cells. Pre-treatment with DTT abolished this interaction, suggesting that PDIA4-mediated inhibition of caspase-3 is redox-dependent (Fig. 4A). To further investigate this mechanism, we generated a caspase-3 mutant by replacing the catalytic cysteine 163 with serine (C163S; Fig. S6A). In cells expressing this mutant and treated with low concentrations of Tm or Tg, PDIA4 formed a stable complex with WT caspase-3 but failed to interact with the C163S mutant (Fig. 4B). The C163S mutation prevented the PDIA4-caspase-3 interaction despite the presence of PDIA4 in the cytosol under these conditions (Fig. 4B and . 1, Figure S1, Figure S6A).

Then we assesed Caspase-3 activity in DTT-treated cells. Under these conditions, the protective effect of Tg or low levels of cisplatin pretreatment, previously shown to confer resistance [27] was lost when cells were exposed to DTT for a short duration at day three of Tg treatment (Fig. 4C–D). These findings correlate with increased proliferation in Tg-pretreated cells, an effect that was not observed in DTT-treated cells (Fig. 4E). Together, these results indicate that cytosolic PDIA4 inhibits caspase-3 through a redox-dependent, cysteine-mediated interaction that requires efficient ER protein reflux.

### 2.5 Redox state determine chaperone-mediated to Bax/Bak-dependent protein reflux

An alternative ER stress-mediated pathway for ER protein reflux to the cytosol has been described, which is facilitated by BAX/BAK and becomes prominent when IRE1 signaling is impaired [12,22]. We therefore investigated whether protein reflux observed under reductive conditions (DTT) or high levels of ER stress (high-dose Tg and Tm), which destabilize DNAJB12 and DNAJB14, proceeds through these BAX/BAK-dependent mechanisms.

**Fig. 5:**
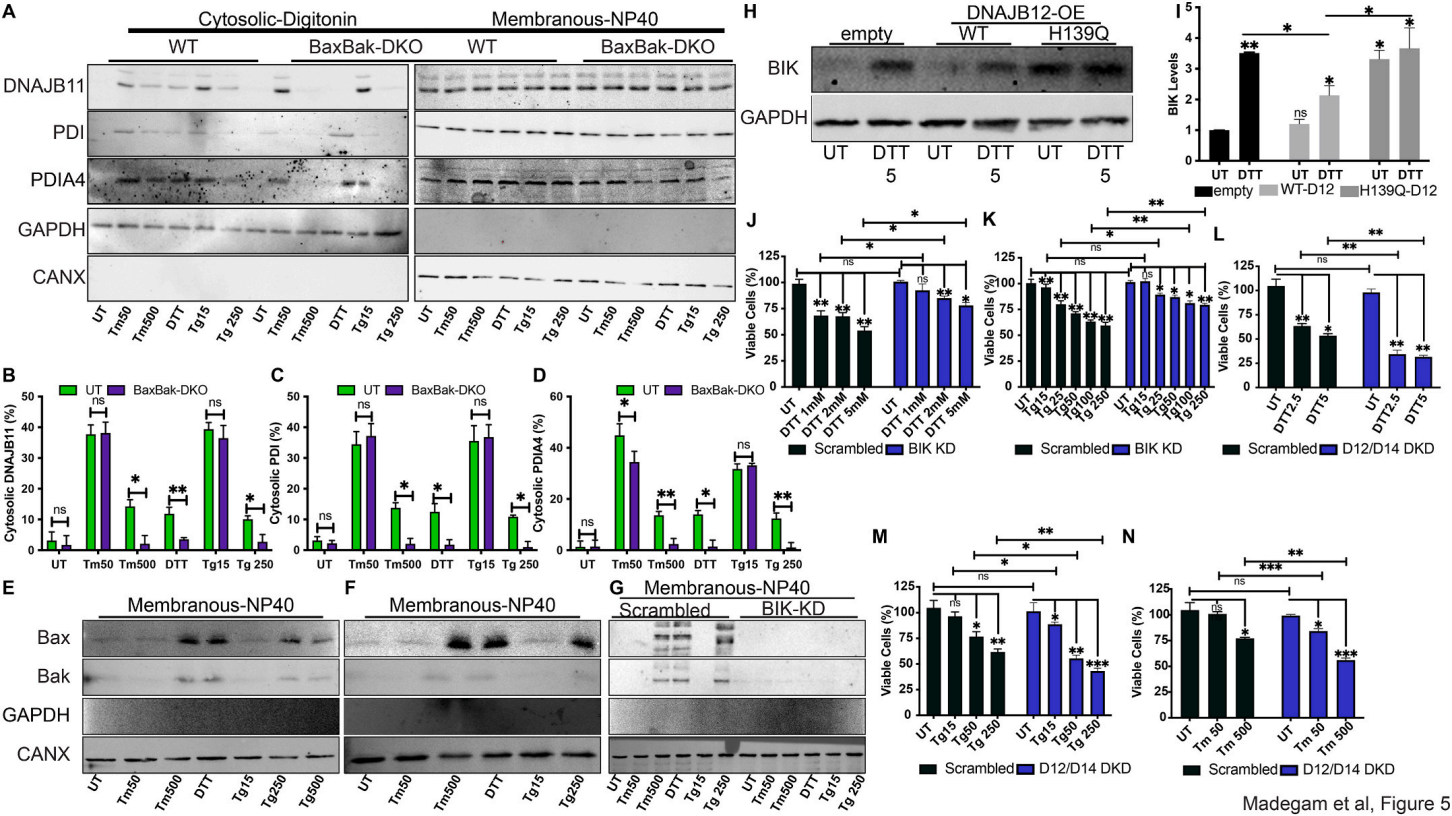
Bax/Bak-dependent protein distribution is triggered under severe ER stress and correlates with cell death. (A) Subcellular protein fractionation showing DNAJB11, PDIA1 (PDI), and PDIA4 in Bax+/+Bak+/+ vs Bax−/−Bak−/− double knockout MEFs in the presence of Tm (ng/mL), Tg (nM), or DTT (mM). N = 3. **(B**–**D)** Quantification of the refluxed ER proteins DNAJB11, PDIA1 (PDI), and PDIA4 as in A, respectively. **(E**–**F)** Representative immunoblot illustrating the localization of Bax and Bak within the ER fraction following treatment with Tm (ng/mL), Tg (nM), or DTT (mM) in A549 and MDA-MB231 cells. N = 3. **(G)** Representative immunoblot showing ER-associated Bax and Bak levels under the same treatments in control (scrambled) versus Bik-silenced cells. N = 3. **(H)** Representative immunoblot showing BIK protein levels in cells overexpressing the WT or H139Q-mutant of DNAJB12 in the presence of DTT. **(I)** Quantification of BIK protein levels as shown in (H). **(J)** Cell viability assay in Bik-silenced A549 cells following treatment with DTT (mM). **(K)** Cell viability assay in Bik-silenced A549 cells after exposure to thapsigargin (Tg). **(L)** Cell viability assay in DNAJB12/14-silenced A549 cells following treatment with DTT (mM). **(M)** Cell viability assay in DNAJB12/14-silenced A549 cells after exposure to thapsigargin (Tg). **(N)** Cell viability assay in DNAJB12/14-silenced A549 cells after exposure to Tunicamycin (Tm). All experiments were performed in biological triplicates. Statistical significance is indicated as follows: *p < 0.05, **p < 0.01, ***p < 0.001.

To address this, we treated mouse embryonic fibroblasts (MEFs) with high concentrations of Tg, Tm, or DTT and monitored ER protein reflux under conditions that promote DNAJB12/14 degradation. Under these conditions, protein reflux persisted (Fig. 5A–D). However, in MEFs lacking both BAX and BAK (Bax/Bak double knockout), the levels of refluxed proteins were markedly reduced (Fig. 5A–D). These findings indicate that under severe or reductive stress, protein reflux shifts from a DNAJB12/14-dependent pathway to a BAX/BAK-dependent mechanism. These data support a model in which cells initially engage a chaperone-mediated reflux pathway to alleviate ER stress. However, when stress is prolonged or exceeds a critical threshold, this adaptive mechanism is suppressed, and cells transition to a BAX/BAK-mediated ER membrane permeabilization pathway associated with apoptotic signaling (Fig. 4C–D).

Mechanistically, we observed that in conditions that decrease DNAJB12 levels, BAX and BAK exhibit increased localization to the ER membrane (Fig. 5E and F). To identify the mediators of this recruitment, we focused on apoptotic proteins that reside on the ER membrane and possess the potential to interact with and recruit BAX and BAK. We identified two candidates: BOK and BIK. BOK is an atypical BCL-2 family protein with established roles in cellular stress responses. While initially thought to function redundantly with BAX and BAK, subsequent studies have shown that BOK can independently permeabilize intracellular membranes and regulate mitochondria-associated membranes (MAMs) by interacting with IP3 receptors, promoting ER-mitochondrial contact sites, and facilitating Ca2+ transfer to the mitochondria [[35], [36], [37]]. BIK is a pro-apoptotic BH3-only protein that localizes to the ER; under severe ER stress, BIK triggeres the activation of initiator caspases and subsequent cell death through effector caspases such as caspase-3 [38].

Because of this, we focused our analysis on BIK as a potential mediator of their recruitment to the ER. We observed that BAX and BAK were recruited to the ER membrane in control cells under severe ER stress, an effect that was abolished in BIK-silenced A549 cells (Fig. 5G and Fig. S7A). indicating that BIK may be the main Bax/Bak recruiter to the ER membrane during ER stress.

Given that DNAJB12 collaborates with Hsp70/Hsc70 to mediate the ERAD of various membrane proteins [39], we hypothesized that BIK might also be a substrate for this complex. To test this, we monitored BIK protein levels upon overexpression of WT DNAJB12 under both basal and DTT-induced stress conditions. In control cells, BIK was undetectable at baseline but increased significantly following DTT treatment. Conversely, overexpression of WT DNAJB12 markedly blunted BIK accumulation under both basal and stress conditions, confirming that DNAJB12 negatively regulates BIK stability during ER stress (Fig. 5H,I and Fig. S7B). To determine whether this regulation depends on the J-domain chaperone activity of DNAJB12, which is required for both its ERAD and ERCYS functions, we overexpressed a J-domain-inactive HPD mutant that is defective in HSP70 interaction and consequently lacks DNAJB12 activity [26,[39], [40], [41]]. Despite being expressed at levels comparable to WT DNAJB12, the HPD mutant markedly stabilized BIK under both basal and DTT-induced stress conditions (Fig. 5H,I and Fig. S7B). The accumulation of BIK in unstressed cells is consistent with the previously reported dominant-negative activity of the HPD mutant [26], indicating that an intact J-domain is required for DNAJB12-mediated BIK turnover. These findings further strengthen the conclusion that DNAJB12 is a key regulator of BIK protein levels.

Because BIK promotes BAX activation at the ER, we next examined whether DNAJB12 affects BAX recruitment to ER membranes. In empty vector-transfected cells, BAX accumulated in the NP40-insoluble membranous fraction following treatment with high concentrations of tunicamycin, thapsigargin, or DTT, consistent with stress-induced recruitment to the ER membrane. In contrast, overexpression of WT DNAJB12 markedly reduced membrane-associated BAX under all stress conditions tested (Fig. S7C–F), suggesting that DNAJB12 suppresses ER stress-induced BAX activation.

These observations were further supported using DNAJB12/DNAJB14 double-knockdown (DKD) cells (Fig. S7G). Similar to the dominant-negative effect of the HPD mutant, BIK was highly stabilized in DKO cells even under basal conditions, demonstrating that endogenous DNAJB12 and DNAJB14 are required to maintain low BIK levels in unstressed cells (Fig. S7H and I). We next examined whether loss of DNAJB12/DNAJB14 affected BAX oligomerization. In untreated cells, BAX was predominantly detected as a monomer, whereas treatment with high concentrations of Tm, Tg, or DTT promoted BAX dimerization (Fig. S7J). Silencing DNAJB12/14 increased the abundance of dimeric BAX even in the absence of stress, and ER stress further enhanced both BAX dimerization and the formation of higher-order oligomers (Fig. S7J), indicating increased activation of the pro-apoptotic pathway.

In summary, our findings identify DNAJB12 and DNAJB14 as key regulators that couple ER protein quality control to cell fate by promoting BIK turnover and limiting BAX activation during ER stress. Rather than acting independently, the DNAJB12/14 and BAX/BAK pathways function in an interconnected manner, with loss of DNAJB12/14 sensitizing cells to stress-induced apoptotic signaling.

To further test our model that BIK mediates ER stress-induced cell death, we assessed cell viability in cells lacking BIK or DNAJB12/14. Loss of BIK significantly reduced apoptosis induced by DTT, tunicamycin, and thapsigargin, resulting in a corresponding increase in cell viability (Fig. 5J and K). These findings demonstrate that BIK is a key mediator of ER membrane permeabilization and downstream apoptotic signaling during unresolved ER stress. In contrast, DNAJB12/DNAJB14 double-knockdown cells were significantly more sensitive to low levels of ER stress, indicating that DNAJB12 and DNAJB14 protect cells under basal and moderate stress conditions (Fig. 5L–N). Together, these findings support a model in which DNAJB12/14 preserve cell survival by limiting BIK accumulation and preventing premature activation of the apoptotic pathway, whereas loss of this protective mechanism sensitizes cells to ER stress-induced cell death.

## 3 Discussion

Cells respond to ER stress by either restoring proteostasis or, when stress exceeds a critical threshold, initiating apoptosis. Here, we identify DNAJB12 and DNAJB14 as redox-sensitive regulators of an adaptive pathway that alleviates ER protein burden through cysteine-dependent regulatory switches. DNAJB12/14-dependent ERCYS operates within a defined stress window, with moderate ER stress promoting maximal protein reflux, whereas severe stress or highly reducing conditions, including those that increase the cellular glutathione pool, disrupt the intramolecular disulfide bonds required for DNAJB12/14 stability. The resulting proteosomal degradation of DNAJB12/14 terminates ERCYS, establishing a biphasic checkpoint that couples the magnitude of ER stress to cell fate decisions.

Our findings identify intracellular glutathione homeostasis as a key regulator of the DNAJB12/14-dependent ERCYS switch, establishing ER redox balance, rather than canonical UPR activation, as the principal determinant of pathway activity. Although BiP/GRP78 is widely used as a marker of ER stress, its expression did not consistently correlate with ERCYS activation at high ER stress conditions, indicating that UPR induction alone is insufficient to predict engagement of this pathway. Instead, our data suggest that the balance between oxidizing and reducing conditions governs ERCYS by controlling the redox-dependent function and stability of DNAJB12 and DNAJB14. Previous work demonstrated that DNAJB12 undergoes oxidative sulfonylation during ER stress, raising the possibility that oxidative modification initially compromises DNAJB12/14 activity, leading to early inhibition of ERCYS [23]. Under sustained or severe oxidative stress, these chaperones are subsequently eliminated through proteasome-dependent degradation, thereby irreversibly terminating the pathway. This sequential mechanism provides a molecular explanation for the transition from adaptive ERCYS-mediated protein reflux to BAX/BAK-dependent ER membrane permeabilization as cellular stress intensifies. Together, these findings establish ERCYS as a regulated, chaperone-driven export pathway whose activation is governed by ER redox homeostasis rather than by ER stress per se. Although the complete repertoire of ERCYS substrates remains to be defined, our results identify cysteine-dependent thiol chemistry as a central mechanism controlling substrate recognition and the efficiency of ER protein reflux.

Beyond the ER membrane, ERCYS continues as an active, chaperone-driven process that requires the cytosolic co-chaperone SGTA. Our data identify SGTA as a critical downstream factor that captures refluxed substrates through cysteine-dependent interactions, likely involving transient disulfide bond formation that stabilizes proteins during their transition into the cytosol. This mechanism is not indiscriminate but highly selective, as specific reactive cysteine residues determine the reflux competence of individual substrates. For example, mutation of the conserved cysteine in AGR2 abolishes its ER exit under conditions that normally activate ERCYS, demonstrating that thiol-dependent interactions contribute directly to substrate selection [26,42].

Once in the cytosol, refluxed proteins can acquire functions distinct from their canonical roles within the ER. We previously demonstrated that cytosolic PDIA4 promotes cell survival in chemoresistant cells by inhibiting caspase-3 [27]. Here, we show that this inhibitory activity depends on a redox-sensitive interaction with the catalytic Cys163 of caspase-3, requiring an oxidizing environment and being abolished under reducing conditions. These findings indicate that ERCYS not only alleviates ER protein burden but also enables the relocalization of selected ER proteins to the cytosol, where they can execute protective, stress-adaptive functions.

These findings raise an important mechanistic question: how can thiol-dependent interactions, which are generally favored in the oxidizing environment of the ER lumen, occur within the reducing cytosol? One possibility is that these reactions are confined to localized redox microdomains with elevated oxidative potential, particularly in the vicinity of the ER membrane, where oxidizing equivalents may transiently diffuse or be generated locally [43,44]. Such microenvironments could be sustained by localized enzymatic activities or dynamic protein assemblies that transiently overcome the globally reducing nature of the cytosol [43,44]. This concept is supported by previous studies showing that ER-resident peroxiredoxin PRDX4 can functionally interact with cytosolic PRDX1 [45], and that ERp46 engage with overoxidized cytosolic PRDX2 [46], demonstrating that redox communication can occur across cellular compartments. Together with our findings, these observations support a model in which localized oxidative environments enable cysteine-dependent substrate capture and redox signaling during ERCYS, despite the overall reducing conditions of the cytosol.

**Fig. 6:**
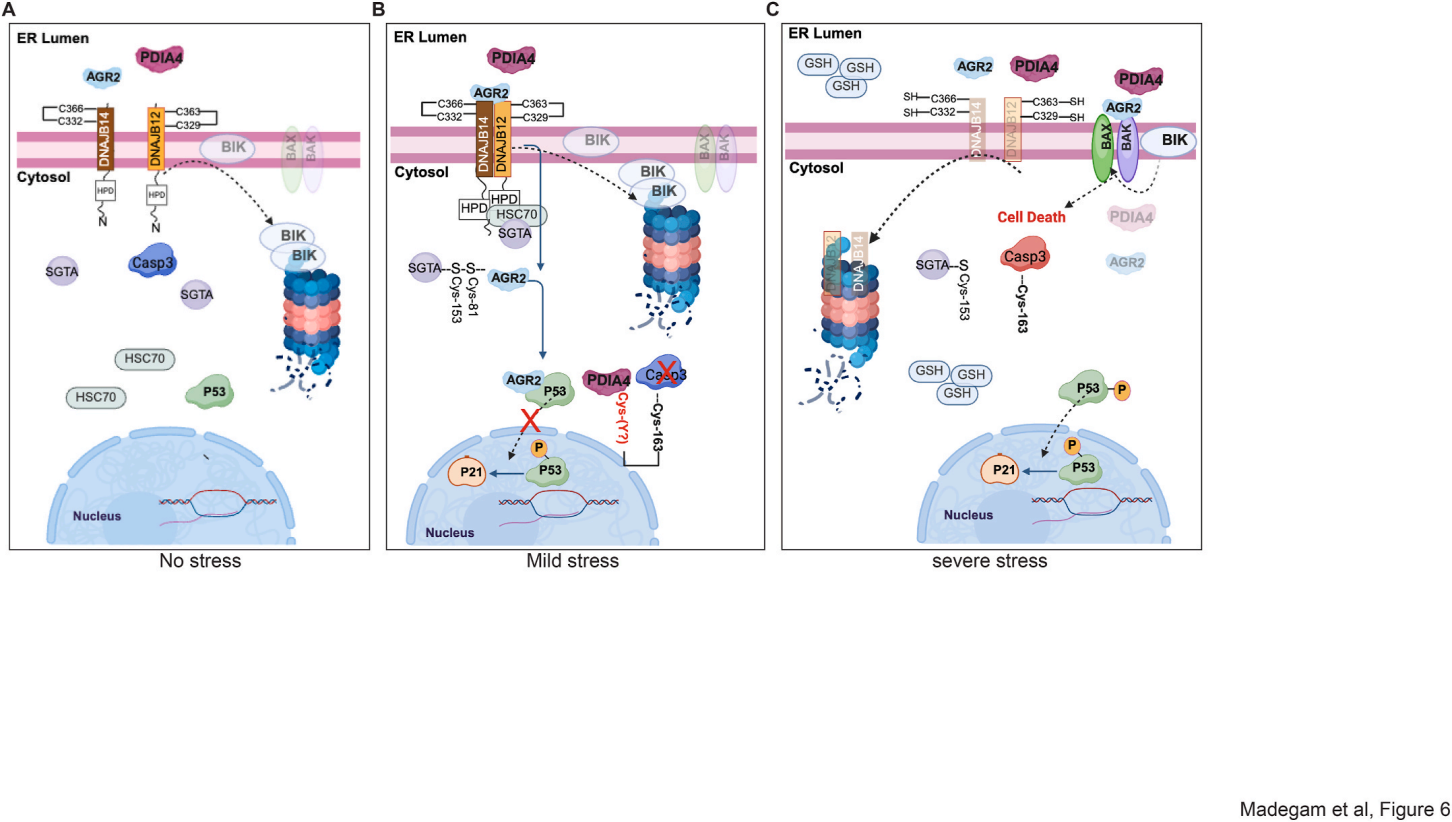
Proposed model illustrating chaperone-mediated and BAX/BAK-dependent pathways of ER protein redistribution. (A) Under unstressed conditions, DNAJB12 and DNAJB14 primarily function in their canonical ER-associated degradation (ERAD) pathway, promoting the proteasomal degradation of ER membrane proteins, including BIK. **(B)** During mild, non-apoptotic ER stress, DNAJB12 and DNAJB14 form a complex with cytosolic chaperones to mediate the reflux of proteins from the ER lumen to the cytosol through a DNAJB12/14-dependent, redox-regulated, chaperone-mediated pathway (ERCYS). At the same time, DNAJB12 facilitates the proteasomal degradation of BIK, preventing the recruitment of BAX and BAK to the ER membrane**. (C)** During severe ER stress or under highly reducing conditions, elevated glutathione levels reduce the intramolecular disulfide bonds of DNAJB12 and DNAJB14, triggering their proteasomal degradation. Loss of DNAJB12/14 abolishes ERCYS and stabilizes BIK, which promotes the recruitment and oligomerization of BAX and BAK at the ER membrane. This results in ER membrane permeabilization and the initiation of apoptotic cell death.

Previous studies have shown that DNAJB12 promotes the degradation of several BCL-2 family proteins, including the atypical family member BOK, highlighting a broader role for DNAJB12/14 in regulating the stability of apoptosis-associated proteins [32,[35], [36], [37]]. Our findings extend this concept by identifying BIK as an additional downstream target of DNAJB12/14-dependent quality control. We propose that, under basal conditions, DNAJB12/14 promote the proteasomal turnover of BIK through a J-domain-dependent mechanism, thereby preventing inappropriate BAX/BAK recruitment to the ER membrane. During severe ER stress, degradation of DNAJB12/14 disrupts this quality control pathway, allowing BIK to accumulate and promote BAX/BAK recruitment, ER membrane permeabilization, and apoptotic signaling. Thus, DNAJB12/14 appear to function as central regulators of ER-associated apoptosis by controlling the stability of multiple pro-apoptotic BCL-2 family proteins. In this model, BIK serves as a key molecular link between the loss of DNAJB12/14 function and activation of the ER-associated apoptotic program (Fig. 6).

Our findings establish DNAJB12/14-dependent ERCYS as a finely tuned, redox-regulated adaptive pathway rather than a simple on/off switch. We propose a two-step model in which moderate ER stress activates ERCYS to alleviate protein burden and restore proteostasis, whereas severe ER stress destabilizes DNAJB12/14, terminating reflux. Importantly, our data indicate that activation of the ER-associated apoptotic pathway requires both severe ER stress and the loss of DNAJB12/14. DNAJB12/14 downregulation stabilizes BIK, but BIK accumulation alone is insufficient to promote BAX/BAK activation. Instead, severe ER stress and DNAJB12/14 loss cooperate to drive BIK-dependent BAX recruitment, oligomerization at the ER membrane, and apoptotic signaling. Thus, the transition from adaptation to apoptosis is governed not by ER stress alone, but by the coordinated collapse of DNAJB12/14-dependent proteostasis and activation of the intrinsic apoptotic machinery.

This transition from adaptive reflux to apoptosis is observed in both pharmacological and physiologically relevant models, including hypoxia-reoxygenation in cardiomyocytes. Under such pathological stress, DNAJB12 degradation coincides with a shift toward BIK- and BAX/BAK-dependent ER membrane permeabilization. We propose that this represents a fundamental molecular switch: analogous to mitochondrial cytochrome *c* release, the ER transitions from a chaperone-mediated survival mode to an apoptotic signaling state upon loss of DNAJB12/14. Collectively, these findings indicate that the core redox-regulated mechanism governing DNAJB12/14 stability, ERCYS, and BAX recruitment is preserved across distinct cellular contexts, independent of PDI expression profiles.

Finally, the DNAJB12/14-dependent, redox-regulated ER protein reflux pathway represents a potential therapeutic vulnerability in cancer. Targeting this pathway, either through inhibition of DNAJB12/14 or by perturbing ER redox homeostasis to disrupt its adaptive function, may enhance ER stress-induced apoptosis and warrants further investigation. Further elucidation of the spatial context of ERCYS, as well as the identification of potential ER-derived apoptotic factors analogous to cytochrome *c*, will be essential for a comprehensive understanding of how thiol chemistry governs the balance between proteostasis and cell death.

## 4 Materials and methods

**Cell culture and reagents.** Cells were cultured under standard tissue culture conditions in a humidified incubator at 37 °C with 5% CO_2_ and atmospheric oxygen. A549 human alveolar basal epithelial adenocarcinoma cells, MDA-MB-231 (M D Anderson-Metastatic Breast-231), human embryonic kidney 293 (HEK293T), mouse embryonic fibroblasts (MEFs), and embryonic BD1X rat heart tissue (H9C2) were used in this study. Cells were maintained in DMEM supplemented with 10% fetal bovine serum (FBS) and penicillin-streptomycin. Phosphate-buffered saline (PBS) was purchased from Hy Laboratories Ltd. Trypsin, FBS, sodium pyruvate, non-essential amino acids (NEAA), l-glutamate, and penicillin-streptomycin were purchased from Gibco. Dithiothreitol (DTT), Tunicamycin (Tm), thapsigargin (Tg), and l-Buthionine-sulfoximine (BSO) were purchased from Sigma-Aldrich.

**Protein isolation and NEM alkylation.** Cells were seeded at a density of 2 × 10^5^ cells/mL in 6-well plates and cultured for 24 h. The medium was then replaced, and cells were treated with Tm (ng/mL), Tg (nM), or DTT (mM) as indicated. Cell pellets were resuspended in Laemmli sample buffer supplemented with N-ethylmaleimide (NEM, 100 mM) and incubated at 37 °C for 30 min. Lysates were centrifuged at 14,000 × g for 10 min at 4 °C, and the supernatants were collected and processed for subsequent analysis.

**Immunoprecipitation (IP).** A549 cells were seeded at a density of 2 × 10^5^ cells/mL. Cells were lysed in IP buffer (150 mM NaCl, 50 mM Tris/HCl, pH 8.0, 0.5% Triton X100, 1 mM EDTA, and 100 mM NEM. Lysates were incubated with specific antibodies against the protein of interest, followed by incubation with protein A/G agarose beads to precipitate protein-antibody complexes. Beads were washed multiple times with PBS containing Tween-20 to remove non-specifically bound proteins. Bound proteins were eluted in Laemmli sample buffer and subsequently analyzed by Western blotting to assess protein-protein interactions.

**Whoe-cell lysate and Western blot.** Cells were rinsed with ice-cold PBS, then lysed by scraping in RIPA buffer. The lysates were centrifuged at 13,000 × g for 10 min at 4 °C, and the clarified supernatant was collected. Protein concentration was measured using the Pierce BCA Protein Assay Kit (Thermo Fisher Scientific) following the manufacturer's protocol. Equal protein concentrations were separated by SDS/PAGE and transferred onto nitrocellulose membranes (Amersham, #10600001). Membranes were blocked with 5% (w/v) non-fat milk prepared in PBS containing 0.1% Tween-20 (PBST) for 1 h at room temperature. They were then incubated overnight at 4 °C with primary antibodies (listed in Supplementary Table S1) diluted in blocking solution. After washing with PBST, membranes were incubated with fluorescent secondary antibodies (listed in Supplementary Table S1). Signal detection was performed using the iBright imaging system (Thermo Fisher Scientific), and band intensities were analyzed using ImageJ software.

**XTT assay:** The XTT assay was performed using the TACS XTT Cell Proliferation Assay Kit (#4891-025-K; R&D Systems) according to the manufacturer's guidelines. Cells (3000 per well) were plated in 96-well plates and treated with ER stressors at the indicated concentrations, as indicated in the figure legends. The XTT working solution was prepared by mixing the XTT labeling reagent with the activator solution. Each well then received 50 μL of the mixture and was incubated for 6 h at 37 °C. Absorbance was recorded at 490 nm with a reference wavelength of 630-690 nm.

**Subcellular protein fractionation:** Separation of cytosolic and membrane-associated proteins was performed as described previously [47,48]. Briefly, cells were detached by trypsinization and collected by centrifugation at 100 × g for 5 min at 4 °C. The cell pellets were washed with ice-cold PBS and centrifuged again under the same conditions. Following the wash step, pellets were resuspended in digitonin-containing buffer (50 mM HEPES, pH 7.4; 150 mM NaCl; 10 μg/mL digitonin [0.001%]) and incubated for 10 min at 4 °C to selectively permeabilize the plasma membrane. Samples were then centrifuged at 2000 × g for 5 min at 4 °C, and the supernatant was collected as the cytosolic (digitonin) fraction, representing soluble proteins not associated with membranes. The remaining pellet was subsequently resuspended in NP-40 lysis buffer (50 mM HEPES, pH 7.4; 150 mM NaCl; 1% NP-40) and incubated on ice for 30 min to extract membrane-associated proteins. After centrifugation at 7000 × g for 5 min at 4 °C, the supernatant was collected as the membranous (NP-40) fraction, which includes proteins localized to or associated with membranous endoplasmic reticulum.

**Caspase-3/7 activity:** The Caspase-Glo 3/7 assay kit (Promega #G8090) was used following the manufacturer's protocol. Briefly, 100 μL of Caspase-Glo 3/7 reagent was added to each well of a 96-well plate and mixed thoroughly. The plate was then incubated at room temperature for 3 h, after which luminescence was measured using a Varioskan Lux microplate reader (Thermo Fisher Scientific).

**Plasmids construction**: Plasmids encoding DNAJB12 and DNAJB14 (WT and HPD mutants) were previously reported by Dabsan et al. [26]. Site-directed mutagenesis was performed to replace cysteine residues with serine using the QuikChange II Site-Directed Mutagenesis Kit (Agilent, #200523). The following primers were used:

C329S-JB12-F:CAACCTCCGGAACAACTcCTGGAAGGAGAAGCAGC.

C329S-JB12-R: GCTGCTTCTCCTTCCAGgAGTTGTTCCGGAGGTTG

C363S-JB12-F:GATGGGCACCCCCAGCTcCAGCCGACTGTCAGAGG.

C363S-JB12-R: CCTCTGACAGTCGGCTGgAGCTGGGGGTGCCCATC

Caspase-3 and SGTA were subcloned into the pcDNA5/FRT/TO vector (Thermo Fisher Scientific) using the primers listed below. For FLAG-tagged constructs, modified forward and reverse primers incorporating the FLAG sequence were used.

Cloning primers for Caspase-3:

Cas3BamF:TAATAAAGGgATCCATGGAGAACACTGAAAACTCAGTGGA

Cas3BamF_Flag:ATAAAGGgATCCATGGACTACAAAGACGATGACGACAAGGAGAACACTGAAAACTCAGTGGATTC

Cas3NotR:CCAACgcggccgcTCTTTAGTGATAAAAATAGAGTTCTTT

Cas3NotR_Flag:CCAACgcggccgcTCTTTACTTGTCGTCATCGTCTTTGTAGTCGTGATAAAAATAGAGTTCTTTTG

Site-directed mutagenesis primers (C163S) for Caspase-3:

Cas3C163SF:TTCATTATTCAGGCCTcCCGTGGTACAGAACTG

Cas3C163SR: CAGTTCTGTACCACGGgAGGCCTGAATAATGAA

Cloning primers for SGTA:

Human SGTA (hSGTA) constructs were generated using primers designed to incorporate epitope tagging and restriction enzyme sites for cloning.

hSGTA-His_F_HB (forward primer; FLAG-tagged hSGTA with *Bam*HI and *Hin*dIII restriction sites):

CACCTCTTaagCTtGGaTCcAGATGGATTACAAGGATGACGACGATAAGGACAACAAGAAGCGCCTGGCCTACGCC

hSGTA_R_Not_Xba (reverse primer; containing *Not*I and *Xba*I restriction sites):

GGTCACAtCtaGAGCGGCcGCGTCACTCCTGCTGGTCGTCGTTGCTGGCGCTGGGCGTCC

Site-directed mutagenesis primers (C153S) for SGTA:

SGTA C153SF: TGTGAGCGGGCCATCTcCATTGACCCGGCCTAC

SGTA C153SR: GTAGGCCGGGTCAATGgAGATGGCCCGCTCACA

Site-directed mutagenesis primers (C81S) for AGR2.

AGR2 C81SF: GATTATTCATCACTTGGATGAGTcCCCACACAGTCAAGCTTT

AGR2 C81SR: AAAGCTTGACTGTGTGGGgACTCATCCAAGTGATGAATAATC

**Hypoxia-reoxygenation protocol:** Cells were exposed to hypoxic conditions (0.5% O_2_) for 8 h in hypoxic chamber. Following hypoxia, the cells were returned to normoxic conditions and reoxygenated for 6 h. ER stressors were added at the onset of the reoxygenation period and maintained throughout the reoxygenation phase.

**Statistical analysis:** Experiments were conducted in three independent biological replicates. Protein band intensities were quantified using ImageJ/Fiji software and cross-checked with measurements obtained from the iBright Imaging System (Thermo Fisher Scientific). Background subtraction was applied uniformly to all blots, and quantification was performed using consistent parameters across experiments. The proportion of cytosolic protein was calculated by dividing the cytosolic fraction signal by the combined signal from both the digitonin and NP-40 fractions. Data analysis was carried out using GraphPad Prism software. Statistical comparisons between groups were made using unpaired, two-tailed Student's t-tests, with significance set at p < 0.05 (*p < 0.01; **p < 0.001).

## Ethics statement

N/A.

## Fundings

This work was supported by the Israel Science Foundation (ISF; Grant No. 977/21) and the Israel Cancer Research Fund (ICRF) Research Career Development Award (RCDA). Laila Abu Madegam was supported by a Kreitman School of Advanced Graduate Studies STEM Fellowship and the Ariane de Rothschild Fellowship. Riafu Adebisi was supported by the Kreitman School of Advanced Graduate Studies.

## CRediT authorship contribution statement

**Laila Abu Madegam:** Conceptualization, Data curation, Formal analysis, Methodology, Validation, Visualization, Writing – original draft, Writing – review & editing. **Noa Gavriel:** Data curation, Formal analysis, Methodology, Validation, Writing – original draft. **Raifu Tolulope Adebisi:** Data curation, Methodology, Validation. **Aeid Igbaria:** Conceptualization, Data curation, Formal analysis, Funding acquisition, Investigation, Methodology, Project administration, Resources, Software, Supervision, Validation, Visualization, Writing – original draft, Writing – review & editing.

## Declaration of competing interest

The authors declare no competing interests.
