# Supplementary material for: DNAJB12/14 redox switching directs chaperone- and Bax/Bak-dependent ER protein reflux
Source: Redox Biol. 2026 Jul 25;96:104324. doi: 10.1016/j.redox.2026.104324 (PMC13445504; doi:10.1016/j.redox.2026.104324)
Supplement: Supplementary file 1 [file mmc1.pdf]

Supplementary information

DNAJB12/14 Redox Switching Directs Chaperone- and Bax/Bak-Dependent ER Protein Reflux

Laila Abu Madegam[1*], Noa Gavriel[1*], Raifu Tolulope Adebisi[1], Aeid Igbaria[1#]

* These authors contributed equally to this work

[1]*The Department of Life Sciences, Ben Gurion University, Beersheba, 841050, Israel.*

[#] Correspondence: aigbaria@bgu.ac.il

Supplementary Materials and Methods:

RNA extraction and Real-Time PCR (qPCR): Total RNA was isolated using the NucleoSpin RNA Mini kit (Macherey-Nagel, #740955.50). 500ng of RNA was reverse transcribed using Maxima™ Reverse Transcriptase (Thermo Fisher Scientific, #EP0742). Quantitative PCR was carried out on a QuantStudio™ 1 Real-Time PCR System (Applied Biosystems, Thermo Fisher Scientific) using 96-well plates. Each reaction mixture (final volume 10 µL) consisted of 10 µL SYBR Green master mix (Thermo Fisher Scientific, #4344463), 1.2 µL forward primer, 1.2 µL reverse primer, 4 µL cDNA, and 3.6 µL nuclease-free water, and was run in technical triplicate with gene-specific primers as previously described [1]. Amplification was performed under standard SYBR Green cycling conditions as specified in the instrument software. ROX dye served as an internal reference. Relative gene expression was determined using the comparative Ct (ΔΔCt) method, with normalization to the housekeeping gene GAPDH and HPRT.

Total glutathione assay: To measure total glutathione, treated cells were washed with cold PBS, scraped into 5% Sulfosalicylic acid (SSA), and frozen in liquid nitrogen. After three freeze-thaw cycles to ensure cell disruption, the mixture was centrifuged at 20,000g for 10 minutes at 4°C to recover the supernatant. For the assay, 5-10 µL of supernatant was added in triplicate to 100 µL of reaction buffer (containing sodium phosphate, EDTA, and glutathione reductase) in a 96-well plate. The reaction was initiated by adding 100 µL of pre-warmed (37°C) buffer containing DTNB and NADPH. Glutathione levels were then determined by monitoring the rate of DTNB reduction via absorbance at 412 nm using a microplate reader (Varioskan LUX, Thermo Scientific).

Bax oligomerization assay: Cells were treated as indicated and incubated overnight. The following day, cells were washed with pre-warmed PBS (37°C) and incubated with 0.1 mM DSP (dithiobis(succinimidyl propionate)) for 30 min at 37°C in a CO_2 incubator to crosslink Bax oligomers. Following incubation, the DSP solution was removed, and cells were rinsed with pre-warmed PBS (37°C). Cells were then incubated with 5 mL of stop solution (20 mM Tris-HCl, pH 7.4 in PBS) at room temperature for 15 min to quench the crosslinking reaction. Proteins were subsequently extracted, and equal amounts of protein lysates were resolved by non-reducing PAGE. Bax oligomerization was detected by immunoblotting using anti-Bax antibodies (Supplementary Table S1).

Supplementary information

Supplementary Table S1:

Antibody (Host)	Company/Cat. No.
DNAJB12 (Rabbit)	Ptg/16780
DNAJB14 (Rabbit)	Ptg/16501
SGTA (Rabbit)	Ptg/11019
SGTA (Mouse)	Ptg/ 60305-1-Ig
PDIA1 (Rabbit)	Ptg/11245-1-AP
BIK	Ptg/68438-1-IG
Bax (6A7)	SantaCruz/sc-23959
Bak (C-3)	SantaCruz/sc-518110
PDIA1 (Mouse)	Ptg/66422-1-Ig
PDIA4 (Rabbit)	Ptg/14712-1-AP
PDIA4 (Mouse)	Ptg/66365-1-Ig
AGR2 (Mouse)	SantaCruz/sc-101211
DNAJB11 (Rabbit)	Ptg/15484-1-AP
FLAG-DYKDDDDK (Mouse)	Ptg/66008-4
GADPH (G-9) (Mouse)	SantaCruz/sc-365062
Goat anti-Mouse IgG (H+L) Highly Cross-Adsorbed Secondary Antibody, Alexa Fluor™ Plus 488	Invitrogen/ #A32723
Goat anti-Rabbit IgG (H+L) Highly Cross-Adsorbed Secondary Antibody, Alexa Fluor™ Plus 488	Invitrogen/ #A32731
Goat anti-Rabbit IgG (H+L) Highly Cross-Adsorbed Secondary Antibody, Alexa Fluor™ Plus 647	Invitrogen/ #A32733
Goat anti-Mouse IgG (H+L) Highly Cross-Adsorbed Secondary Antibody, Alexa Fluor™ Plus 647	Invitrogen/ #A32728
Goat anti-Rabbit IgG (H+L) Highly Cross-Adsorbed Secondary Antibody, Alexa Fluor™ Plus 555	Invitrogen/ #A32732
Goat anti-Rat IgG (H+L) Cross-Adsorbed Secondary Antibody, Alexa Fluor™ 488	Invitrogen/ #A-11006
Goat anti-Rat IgG (H+L) Highly Cross-Adsorbed Secondary Antibody, Alexa Fluor™ Plus 647	Invitrogen/ #A48265

Table Legends:

Supplementary Table S1: List of antibodies used in this study

Supplementary information

Figure Legends:

Supplementary Figure S1: (A) Subcellular protein fractionation (Cytosolic-Digitonin fraction) of DNAJB11, PDIA1 (PDI), and PDIA4 in MDA-MB-231 cells treated with Tm (ng/mL), Tg (nM), and DTT (mM). N=3. **(B-D)** Quantification of the refluxed ER proteins DNAJB11, PDIA1 (PDI), and PDIA4 as in A, respectively. **(E-G)** qPCR of relative mRNA levels of the IRE1-regulated genes XBP1s, PERK regulated gene GADD34, and ATF6 regulated gene BIP respectively in A549 cells treated with different concentrations of Tm (ng/mL), Tg (nM), and DTT (mM). N=3. **(H-J)** qPCR of relative mRNA levels of the IRE1-regulated genes XBP1s, PERK regulated gene GADD34, and ATF6 regulated gene BIP respectively in MDA-MB-231 cells treated with different concentrations of Tm (ng/mL), Tg (nM), and DTT (mM). N=3. **(K)** Subcellular protein fractionation (Cytosolic-Digitonin fraction) of DNAJB11, PDIA1 (PDI), and PDIA4 in HEK293T cells treated with Tm (ng/mL), Tg (nM), and DTT (mM). N=3. **(L-N)** Quantification of the refluxed ER proteins DNAJB11, PDIA1 (PDI), and PDIA4 as in K, respectively. **(O)** Subcellular protein fractionation (Cytosolic-Digitonin fraction) of DNAJB11, PDIA1 (PDI), and PDIA4 in H9C2 cells under hypoxia/reoxygenation (H/R) and Tm treatment. **(P-R)** Quantification of the refluxed ER proteins DNAJB11, PDIA1 (PDI), and PDIA4 as in O, respectively. **(S)** qPCR of relative mRNA levels in H9C2 cells under H/R and Tm treatment. All experiments were conducted in biological triplicates. Statistical significance is denoted as follows: *$p < 0.05$, **$p < 0.01$, ***$p < 0.001$.

Supplementary Figure S2: (A) Fold change in caspase-3/7 activity in A549 cells treated with different concentrations of Tm (ng/mL), Tg (nM), and DTT (mM). N=3. **(B)** XTT viability assay in A549 cells treated with different concentrations of Tm (ng/mL), Tg (nM),

and DTT (mM). N=3. **(C-D)** Quantification of SGTA(C) and HSC70(D) as shown in main Figure 1E and normalized to GAPDH N=3. **(E)** Representative immunoblot of DNAJB12, DNAJB14, and GAPDH in HEK293T cells treated with Tm (ng/mL), Tg (nM), and DTT (mM) in the presence of the proteasome inhibitor MG-132. **(F-G)** Quantification of DNAJB12(F) and DNAJB14(F) as shown in E and normalized to GAPDH N=3. All experiments were conducted in biological triplicates. Statistical significance is denoted as follows: *p < 0.05, **p < 0.01, ***p < 0.001.

Supplementary Figure S3: (A) Subcellular fractionation (digitonin-extracted cytosolic fraction) showing DNAJB11, PDIA1 (PDI), and PDIA4 in A549 cells pre-treated with DTT prior to exposure to Tm (ng/mL), Tg (nM), or DTT (mM). N=3. **(B-D)** Quantification of the refluxed ER proteins DNAJB11, PDIA1 (PDI), and PDIA4 as in A, respectively. **(E)** Subcellular fractionation (digitonin-extracted cytosolic fraction) showing DNAJB11, PDIA1 (PDI), and PDIA4 in MDA-MB-231 cells pre-treated with DTT prior to exposure to Tm (ng/mL), Tg (nM), or DTT (mM). N=3. **(F-H)** Quantification of the refluxed ER proteins DNAJB11, PDIA1 (PDI), and PDIA4 as in E, respectively. All experiments were conducted in biological triplicates. Statistical significance is denoted as follows: *p < 0.05, **p < 0.01, ***p < 0.001.

Supplementary Figure S4: (A) Representative immunoblots from two independent experiments showing NEM-labeled DNAJB12 and DNAJB14 in A549 cells under mild and severe ER stress conditions, resolved under reducing and non-reducing conditions as indicated. N=3. **(B)** Representative immunoblots from two independent experiments showing expression levels of FLAG-tagged DNAJB12 (WT, C329S, and C363S) compared to empty vector controls. **(C)** Quantitative analysis of protein expression levels

shown in (B). **(D)** Cell viability assay in A549 cells expressing FLAG-tagged DNAJB12 (WT, C329S, and C363S) under a doxycycline-inducible system. N=3. **(E)** Cellular GSH levels in A549 cells under mild and severe ER stress conditions. **(F)** GSH/GSSG ratio in A549 cells under mild and severe ER stress conditions. **(G)** qPCR of relative BIP mRNA levels in A549 cells treated with different concentrations of Tm (ng/mL), Tg (nM), and DTT (mM). N=3. All experiments were conducted in biological triplicates. Statistical significance is denoted as follows: *p < 0.05, **p < 0.01, ***p < 0.001.

Supplementary Figure S5: (A-B) Representative immunoblots from two independent experiments showing FLAG-tagged SGTA expression in cells expressing either the wild-type protein or the C153S cysteine mutant. **(C)** Subcellular fractionation (digitonin-extracted cytosolic fraction) showing FLAG-AGR2(C81S) and PDIA4 in A549 cells under low (L) and high (H) ER stress conditions. N=3. **(D-E)** Quantification of the refluxed ER proteins FLAG-AGR2(C81S) and PDIA4 as in C, respectively. All experiments were conducted in biological triplicates. Statistical significance is denoted as follows: *p < 0.05, **p < 0.01, ***p < 0.001.

Supplementary Figure S6: (A) Representative immunoblot showing FLAG-tagged caspase-3 expression in cells expressing either the wild-type protein or the C163S cysteine mutant.

Supplementary Figure S7: (A) Representative immunoblot showing BIK protein levels in BIK silenced A549 cells. **(B)** Representative immunoblot showing FLAG-tagged DNAJB12 WT and H139Q protein levels in A549 cells. **(C)** Representative immunoblot showing ER-associated Bax levels under under low and high ER stress conditions. N=3.

(D) Quantification of ER-associated Bax levels as shown in C. **(E)** Representative immunoblot showing ER-associated Bax levels in HEK293T cells iverexpressing WT-DNAJB12. N=3**.** **(F)** Quantification of ER-associated Bax levels as shown in E. **(G)** Representative immunoblot showing DNAJB12 and DNAJB14 protein levels in DNAJB12/DNAJB14 silenced A549 cells. **(H)** Representative immunoblot showing BIK protein levels in DNAJB12/DNAJB14 silenced A549 cells**.** **(I)** Quantification of BIK protein levels in DNAJB12/DNAJB14 silenced A549 cells. **(J)** Bax oligomerization state in control and DNAJB12/DNAJB14 silenced A549 cells upon treatment with high ER stress conditions. All experiments were conducted in biological triplicates. Statistical significance is denoted as follows: *p < 0.05, **p < 0.01, ***p < 0.001.

Supplementary References:

1. Sicari, D., et al., *A guide to assessing endoplasmic reticulum homeostasis and stress in mammalian systems*. FEBS J, 2020. **287**(1): p. 27-42.

Supplementary information

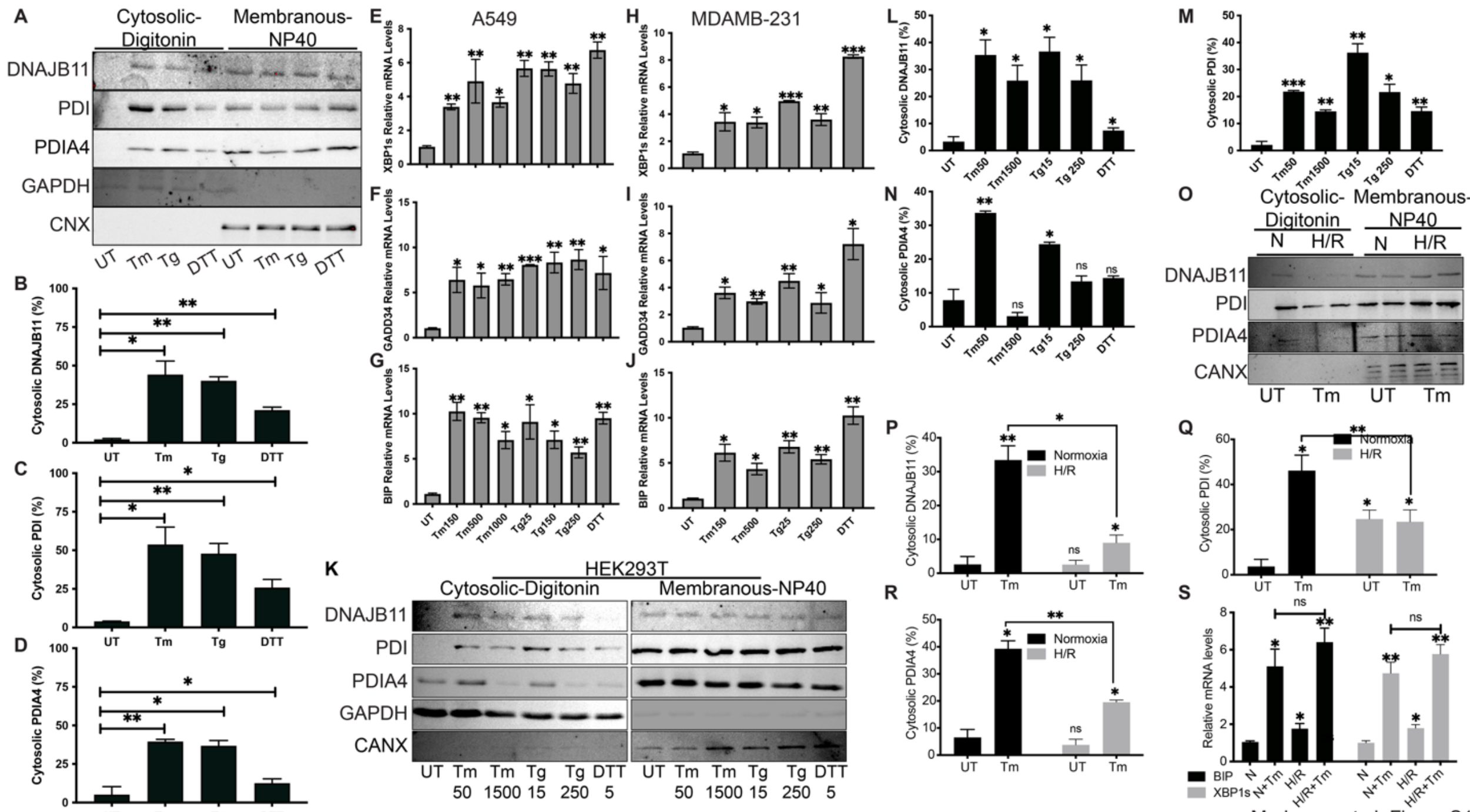

Madegam et al, Figure S1

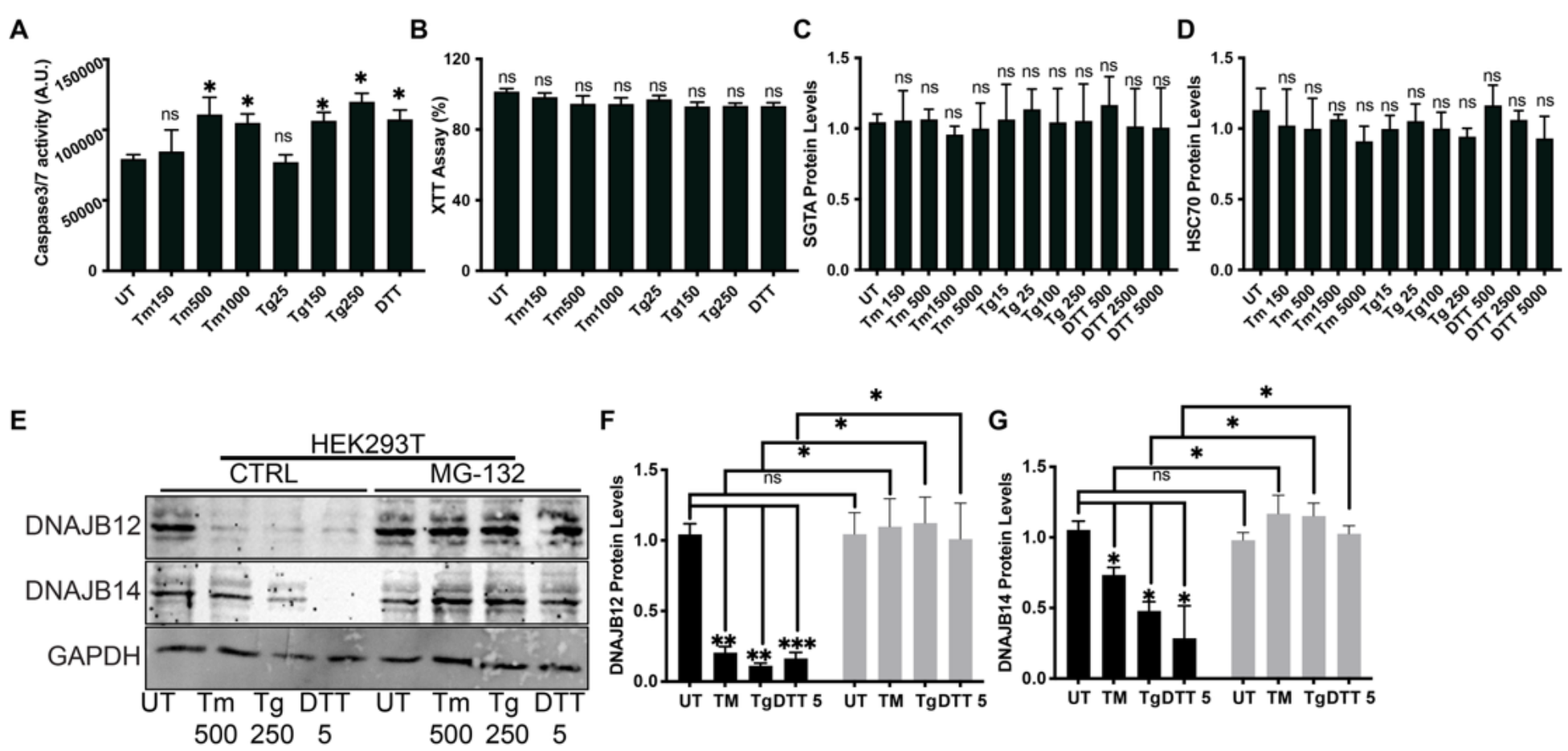

Madegam et al, Figure S2

Supplementary information

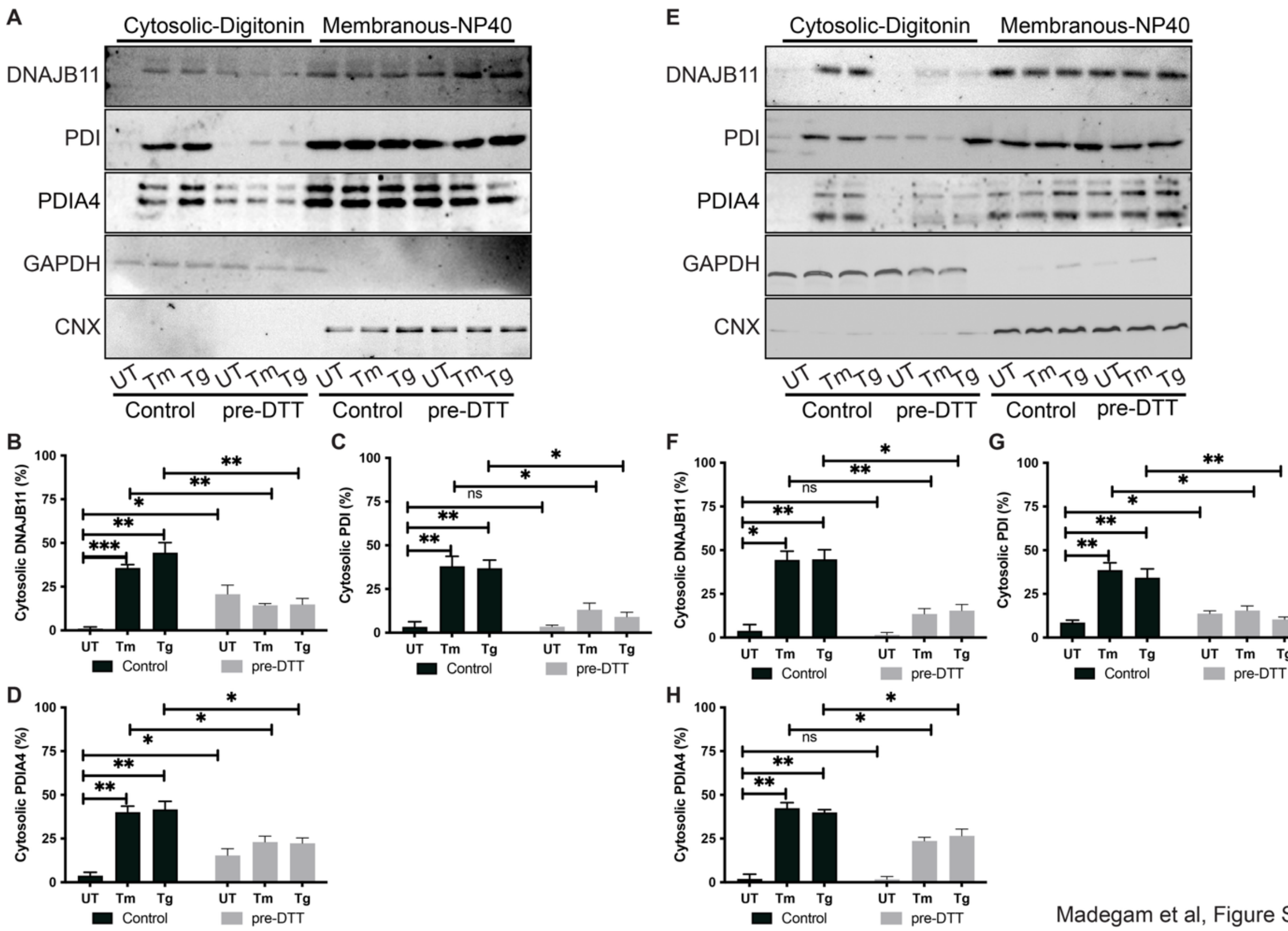

Madegam et al, Figure S3

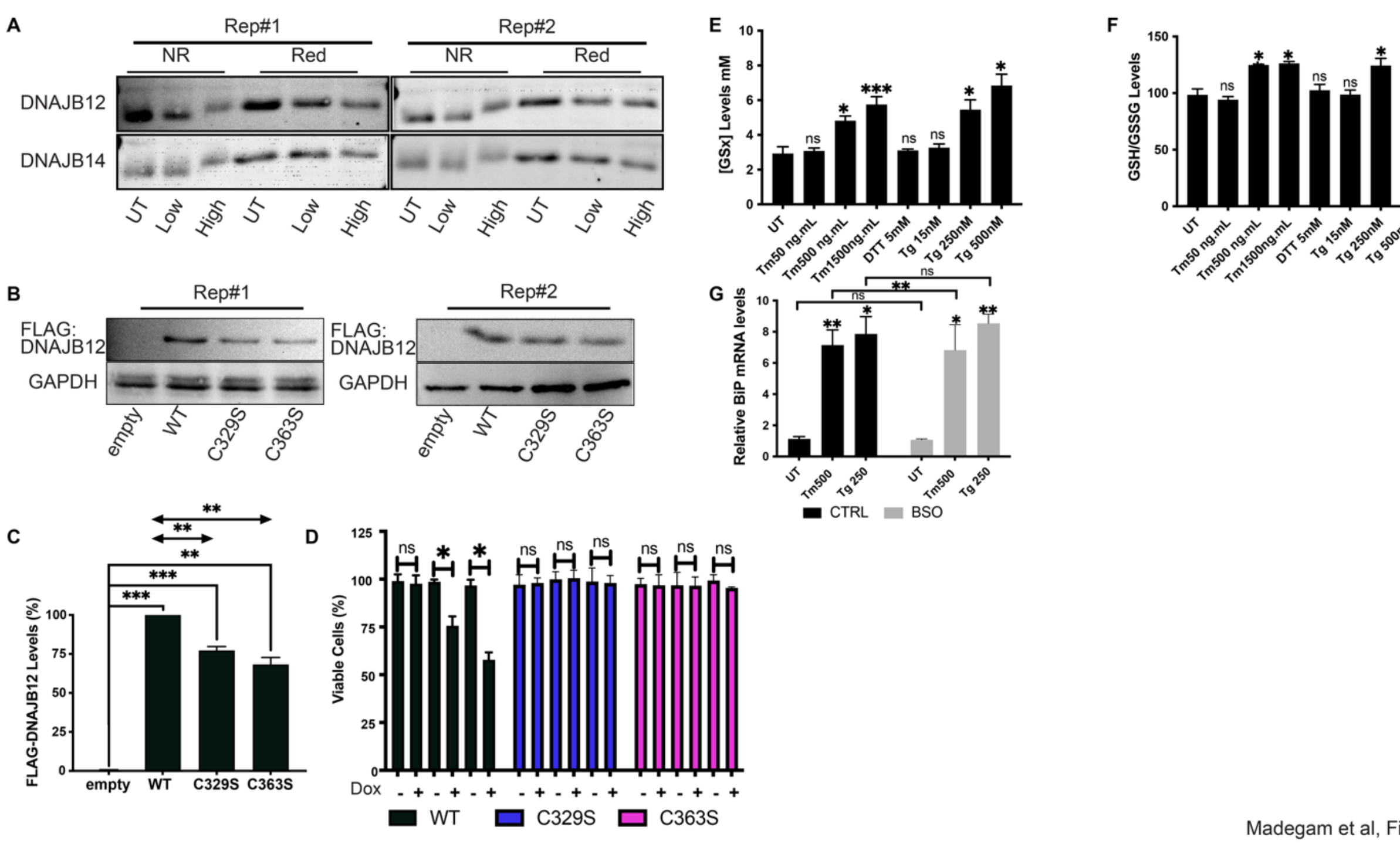

Madegam et al, Figure S4

Supplementary information

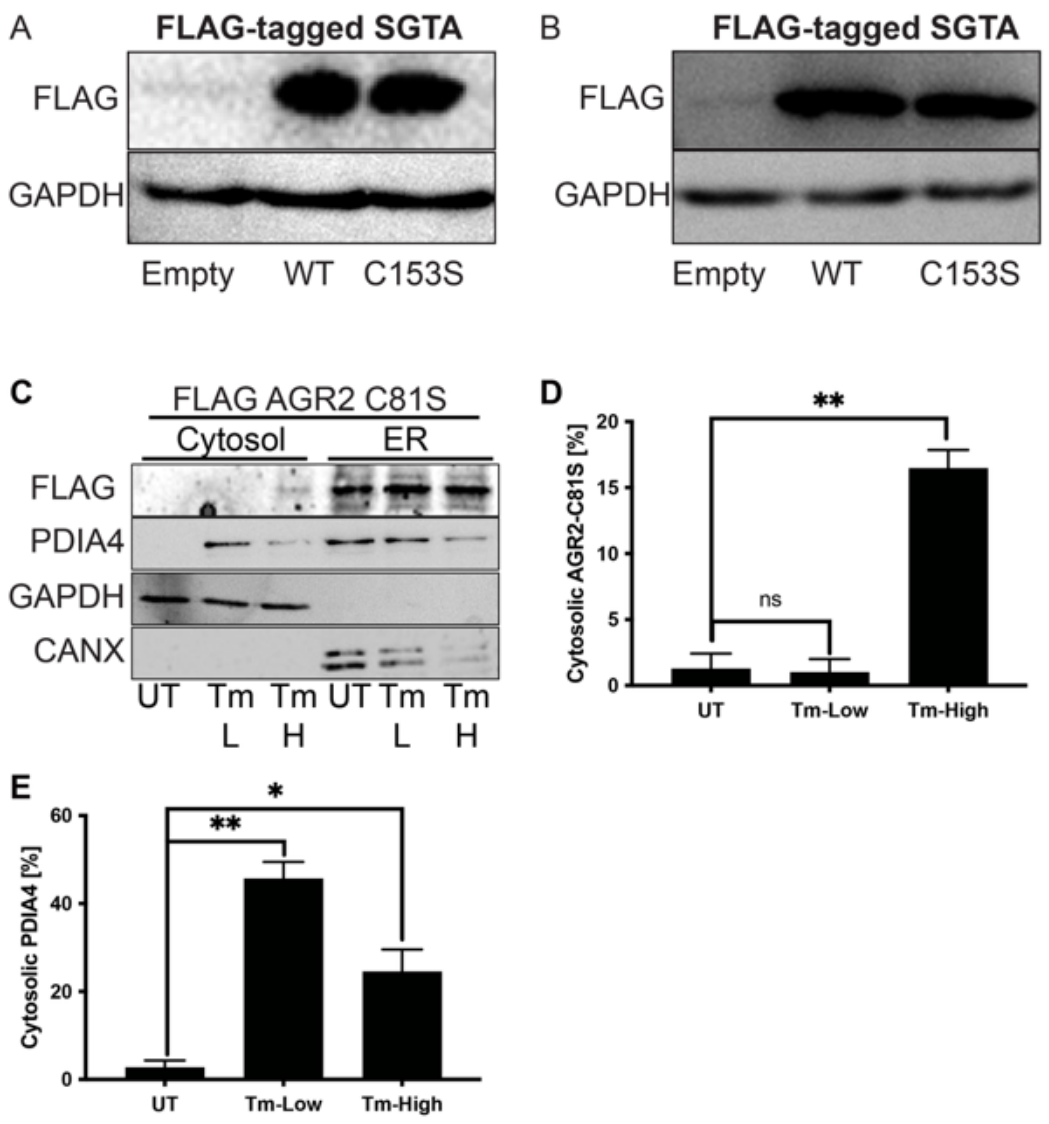

Madegam et al, Figure S5

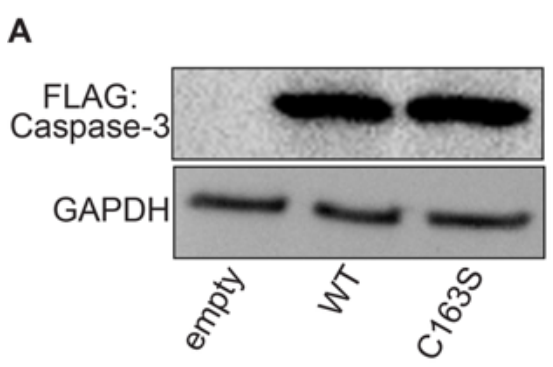

Madegam et al, Figure S6

Supplementary information

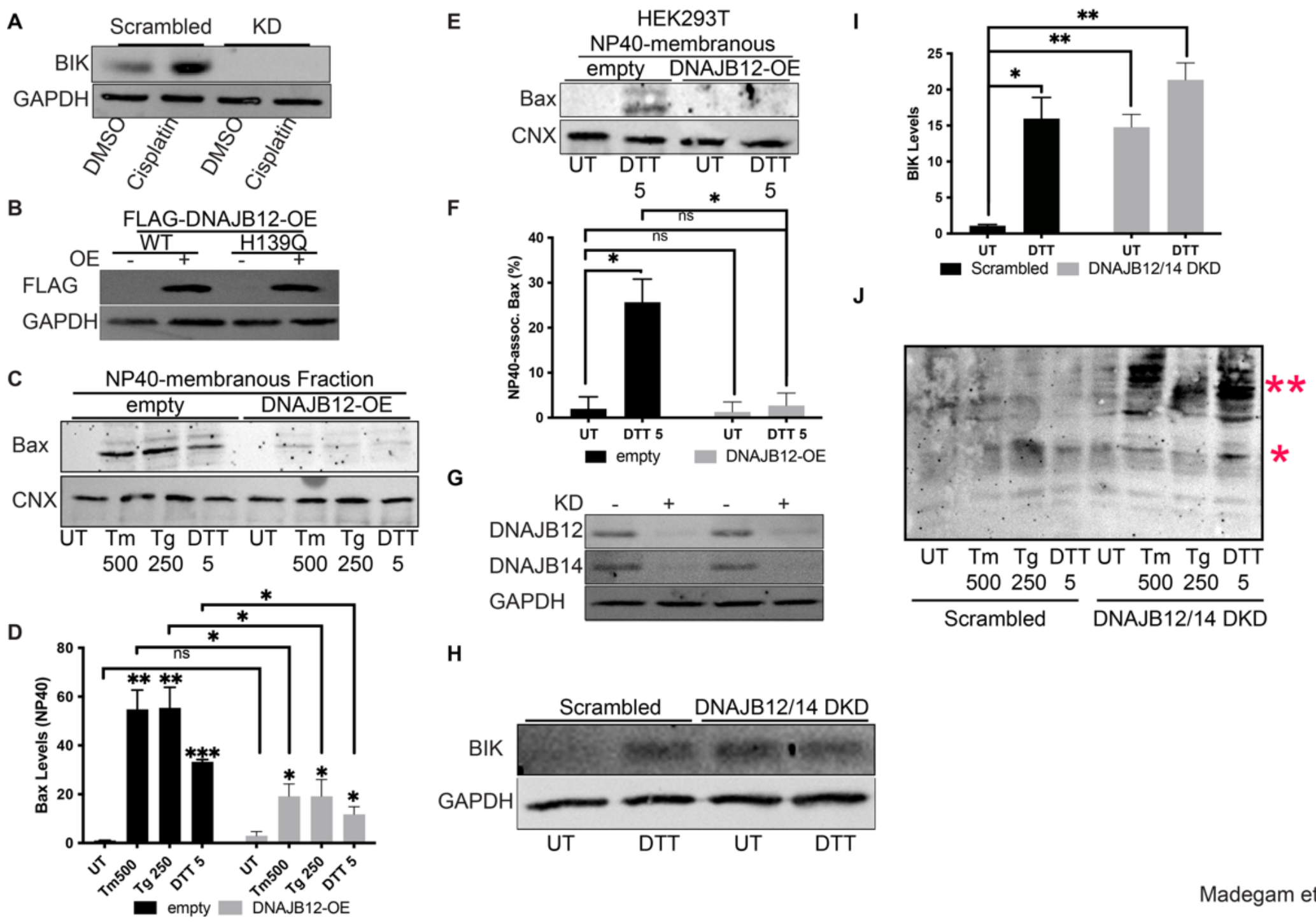

Madegam et al, Supplementary Figure S7
